# Outcomes of a Comprehensive Mobile Vaping Cessation Program in Adults Who Vape Daily: Cohort Study

**DOI:** 10.2196/57376

**Published:** 2024-10-28

**Authors:** Jennifer D Marler, Craig A Fujii, MacKenzie T Utley, Daniel J Balbierz, Joseph A Galanko, David S Utley

**Affiliations:** 1 Pivot Health Technologies, Inc San Carlos, CA United States; 2 Department of Pediatrics University of North Carolina Chapel Hill, NC United States

**Keywords:** app, digital health, mobile health, mHealth, mobile apps, smartphone, vaping, vaping cessation, mobile phone

## Abstract

**Background:**

In the United States, e-cigarettes, or vapes, are the second most commonly used tobacco product. Despite abundant smartphone app–based cigarette cessation programs, there are few such programs for vaping and even fewer supporting data.

**Objective:**

This exploratory, prospective, single-arm, remote cohort study of the Pivot vaping cessation program assessed enrollment and questionnaire completion rates, participant engagement and retention, changes in attitudes toward quitting vaping, changes in vaping behavior, and participant feedback. We aimed to establish early data to inform program improvements and future study design.

**Methods:**

American adults aged ≥21 years who vaped daily, reported ≥5 vape sessions per day, and planned to quit vaping within 6 months were recruited on the web. Data were self-reported via app- and web-based questionnaires. Outcomes included engagement and retention (ie, weeks in the program, number of Pivot app openings, and number of messages sent to the coach), vaping attitudes (ie, success in quitting and difficulty staying quit), vaping behavior (ie, quit attempts, Penn State Electronic Cigarette Dependence Index, 7- and 30-day point-prevalence abstinence [PPA], and continuous abstinence [defined as ≥7-day PPA at 12 weeks+30-day PPA at 26 weeks+0 vaping sessions since 12 weeks]), and participant feedback.

**Results:**

In total, 73 participants onboarded (intention-to-treat sample); 68 (93%) completed the 12- and 26-week questionnaires (completer samples). On average, participants were active in Pivot for 13.8 (SD 7.3) weeks, had 87.3 (SD 99.9) app sessions, and sent 37.6 (SD 42.3) messages to their coach over 26 weeks. Mean success in quitting and difficulty staying quit (scale of 1-10) improved from baseline to 12 weeks—4.9 (SD 2.9) to 7.0 (SD 3.0) and 4.0 (SD 2.8) to 6.2 (SD 3.1), respectively (*P*<.001 in both cases). Most participants (64/73, 88%) made ≥1 quit attempt. At 26 weeks, intention-to-treat 7-day PPA, 30-day PPA, and continuous abstinence rates were 48% (35/73), 45% (33/73), and 30% (22/73), respectively. In total, 45% (33/73) of the participants did not achieve 7-day PPA at 26 weeks; their mean Penn State Electronic Cigarette Dependence Index score decreased from baseline (13.9, SD 3.1) to 26 weeks (10.8, SD 4.5; mean change –3.2, SD 3.9; *P*<.001); 48% (16/33) of these participants improved in the e-cigarette dependence category. At 2 weeks, 72% (51/71) of respondents reported that using Pivot increased their motivation to quit vaping; at 4 weeks, 79% (55/70) reported using Pivot decreased the amount they vaped per day.

**Conclusions:**

In this first evaluation of Pivot in adult daily vapers, questionnaire completion rates were >90%, average program engagement duration was approximately 14 weeks, and most participants reported increased motivation to quit vaping. These and early cessation outcomes herein suggest a role for Pivot in vaping cessation and will inform associated future study and program improvements.

## Introduction

### Background

e-Cigarette use, or vaping, has become a significant public health problem. In 2022, a total of 6% of American adults were e-cigarette users [1]. e-Cigarettes remain the second most used tobacco product behind cigarettes, and many e-cigarette users are current or former smokers [2].

The long-term health consequences associated with vaping are not well understood due to the relatively new nature of e-cigarettes and the variety of e-liquids that are consumed. However, e-cigarette liquids commonly contain compounds linked to cancer and serious lung disease, and improper use of these devices can cause unintended bodily injury [3]. Thus, quitting e-cigarette use is an important step for a user’s health and safety. Adult e-cigarette users in the United States continue to vape despite being interested in quitting [4,5], demonstrating a need for additional vaping cessation programs with resources and support to guide these individuals through successful quit attempts.

Of the limited research on digital vaping cessation programs, 4 studies have outcomes specific to vaping cessation. A 2023 feasibility study by Webb et al [6] reported preliminary outcomes of the Quit Genius-Vaping tobacco cessation program, a smartphone app based on cognitive behavioral therapy with nicotine replacement therapy (NRT) and coaching. At 1 month after the quit date (approximately 5-6 weeks after program enrollment), 7-day point-prevalence abstinence (PPA) was achieved by 43% (22/51; intention to treat [ITT]) of the participants, and 30-day PPA was achieved by 25% (13/51; ITT) of the participants [6].

Another feasibility study conducted in 2022 by Palmer et al [7] reported preliminary vaping outcomes for participants randomized to an SMS text message–based cessation program (“This is Quitting”) with contingency management (n=22) or the SMS text message–based cessation program with monitoring control (n=5). At 1 month after treatment (day 56), 27% (6/22) in the contingency management intervention group and 20% (1/5) in the control group self-reported vaping abstinence since the end of treatment, between days 28 and 56 of the study [7].

A 2021 randomized controlled trial (RCT) by Graham et al [8] investigated the This is Quitting SMS text messaging program in young adults. US-based, past–30-day e-cigarette users (n=1304) were compared to individuals randomized to an assessment-only control arm (n=1284). Of the participants randomized to the This is Quitting program, 24.08% (314/1304; ITT; 95% CI 21.8-26.5) achieved 30-day PPA at 7 months, more than 18.61% (239/1284; ITT; 95% CI 16.7-20.8) in the control assessment-only group (odds ratio 1.39, 95% CI 1.15-1.68; *P*<.001) [8].

Another 2021 study investigated the combination of NRT and behavioral support (n=7) compared to vape tapering with behavioral support (n=8) and a self-guided program alone (n=9) among adults who currently vaped. At 6 months, 43% (3/7; ITT) of the participants in the NRT and behavioral support group were vape and nicotine free compared to 75% (6/8; ITT) in the vape tapering and behavioral support group and 44% (4/9; ITT) in the self-guided program group [9]. However, this study is limited due to its small sample size and the fact that 6-month data were collected from only 16 participants.

While these 4 studies provide important early data on digital interventions for vaping cessation, their small sample sizes, except for that of the study by Graham et al [8], and the overall scarcity of vaping intervention publications in the literature demonstrate the need for additional investigation in this area. This study on the Pivot vaping cessation program aimed to help address this need.

Historically, Pivot’s tobacco cessation program has focused on smoking cessation [10-15]. This experience, along with recent changes to the tobacco use landscape, made the program well suited to expand to e-cigarettes. Similar to its smoking cessation counterpart, the Pivot vaping cessation program is comprehensive and evidence based. It includes a unique combination of app-based educational content; interactive features such as games, self assessments, and vape use tracking; in-app messaging-based human coaching; and a web-based community discussion forum, all tailored to vaping. Considering the novelty of this approach and the lack of associated investigation to date, this initial study of Pivot for vaping cessation starts to address the evidence gap by assessing participant retention and engagement, changes in vaping attitudes and behavior, and feedback.

### Objectives

This first clinical study of the Pivot vaping cessation program aimed to assess the program in adults who vape daily so as to establish early data to inform program improvements and future study design.

## Methods

### Study Design

This was a prospective open-label, single-arm study of the Pivot vaping cessation program (Pivot Health Technologies Inc). This remote study was conducted as an exploratory study to assess the initial performance of Pivot for vaping cessation. This study examined participant retention and engagement, changes in attitudes toward quitting vaping, changes in vaping behavior, and participant feedback on the program.

### Ethical Considerations

All participants provided electronic informed consent before taking part. The study was reviewed and approved by Solutions Institutional Review Board, LLC (protocol 2022/11/10), and registered at ClinicalTrials.gov (NCT05642598). Study data were imported directly into a secure database (PostgreSQL; PostgreSQL Global Development Group). Participants were compensated with US $25 to US $50 per completed study questionnaire for up to a total of US $250 for 8 questionnaires over the 26-week study period. Compensation was in the form of Visa or Mastercard gift cards that were mailed or emailed to participants approximately 2 to 3 weeks after completing the associated questionnaire or questionnaires. Compensation was not associated with use of the various components of Pivot, level of engagement, or vaping or tobacco use status. Payments were bundled, with participants receiving up to 4 payments over the 26-week course of the study.

### Participants

Eligibility criteria were being aged ≥21 years, daily nicotine vape use for at least the previous 30 days, planning to quit vaping in the next 6 months, ≥5 average vape sessions per day (VSPD), interest in working with a vape cessation coach via SMS text messaging, self-reported abstinence from cigarettes for at least the previous 3 months for participants who were former cigarette smokers, being a resident of the United States, being able to read and comprehend English, owning and using a smartphone compatible with the study app (iPhone 5 and above with operating system iOS 12 and above or Android 7.0 and above with operating system Android 7.0 and above), and having daily internet access on their smartphone and self-reporting comfort with downloading and using smartphone apps. The eligibility criteria of being aged ≥21 years was included because 21 is the minimum age to purchase e-cigarettes in the United States and because the current version of the Pivot vaping cessation program is designed for individuals in this age group (≥21 years). The eligibility criteria of ≥5 average VSPD was arbitrary and an attempt to enroll individuals who vaped regularly rather than sporadically.

Exclusion criteria were current use of other vaping cessation apps, coaching, classes, or quit programs; current use of cigarettes; failure to provide contact or collateral information or verify email address; and participation in a previous study sponsored by Pivot Health Technologies Inc. As this was not an RCT, participants were not blinded, and there was no control group.

### Recruitment

Participants were recruited in the United States through online media (Facebook, Google Ads, Reddit, and Craigslist). Potential participants were asked to provide contact information and answer questions on demographics and tobacco use using a web-based screening form. Staff followed the study protocol for specific review criteria—web-based screening form submissions were reviewed in the order received, and potential participants meeting initial eligibility requirements were then called on a first-come-first-serve basis, with nonproportional quota sampling enrollment guidelines applied.

These nonproportional quota sampling guidelines included the following: enrolling 40% to 60% of participants who had never been cigarette smokers, 40% to 60% of participants who started vaping in an effort to quit smoking, no more than 60% of participants from any self-identified gender group, no more than 50% of participants from any decade-spanning age group (eg, 30 to 39 years), no more than 70% of participants identifying as White individuals, and up to 20% of participants not employed. The overarching goal of these nonproportional quota sampling ranges was to ensure representation among genders, racial and ethnic minority groups, age groups, and individuals of varying socioeconomic statuses.

Specifically, we included the nonproportional quota sampling goals related to history of cigarette smoking to align with characteristics of adult e-cigarette users in the United States who are not current cigarette smokers—approximately 60% are former smokers, and 40% are never smokers [16,17]. Targeting no more than 60% of participants from any self-identified gender group was to align with the gender breakdown of American adults and prevalence of e-cigarette use among these gender groups [18,19]. Pursuing <50% of participants in any particular decade-spanning age group was arbitrary and an attempt to avoid overrepresentation of any age group. The goal of no more than 70% of participants identifying as White individuals was similar to goals implemented in other studies assessing digital interventions in tobacco users [20,21] and in acknowledgment that individuals identifying as belonging to racial and ethnic minority groups are underrepresented in research studies [22,23]. Inclusion of up to 20% of participants who were not employed reflects that, currently, Pivot is primarily offered through employers or health insurance providers while also ensuring representation of people who do not receive compensation for work (ie, stay-at-home parents, students, care providers, retired individuals, and individuals who are otherwise not employed).

### Eligibility

During the screening phone call, potential participants were asked questions to confirm study eligibility. Study personnel informed the potential participants of the study details and answered any questions. Potential eligible participants who wanted to proceed with the study were emailed an electronic Health Insurance Portability and Accountability Act authorization form and an electronic informed consent form, which they signed before participating in the study.

### Onboarding

Upon providing electronic informed consent, participants completed the web-based baseline questionnaire, which collected information on demographics, vaping and smoking history and current status, and attitudes toward quitting vaping (ie, readiness to quit, confidence to quit, and perceived difficulty of quitting). Participants were provided instructions to download the Pivot program to their smartphone. Getting started in Pivot was a self-guided process. Participants had access to customer service and study staff as needed. After successful onboarding, they were paired with a live, dedicated tobacco cessation coach who provided one-on-one support over the course of the study.

### Pivot Vaping Cessation Program

Pivot is a 12-month app-based vaping cessation program. Users are provided with 12 months of full program access to ensure that all have the option to engage with a coach and the community over this period, acknowledging that most users will be done using the program by 6 months. The program was developed and designed primarily for delivery in the context of employee wellness programs and health plans.

Pivot is based on the US clinical practice guideline for tobacco cessation and leverages evidence-based principles and clinical best practices [24]. Specifically, Pivot uses the 5 A’s (Ask, Advise, Assess, Assist, and Arrange); tailors to readiness to quit through user selection of associated program pathways [24]; capitalizes on effective methods for tobacco cessation (eg, motivational interviewing, cognitive behavioral therapy, and self-determination theory) [25-29]; and provides behavioral counseling through a live, dedicated coach [24,30,31].

**Figure 1:**
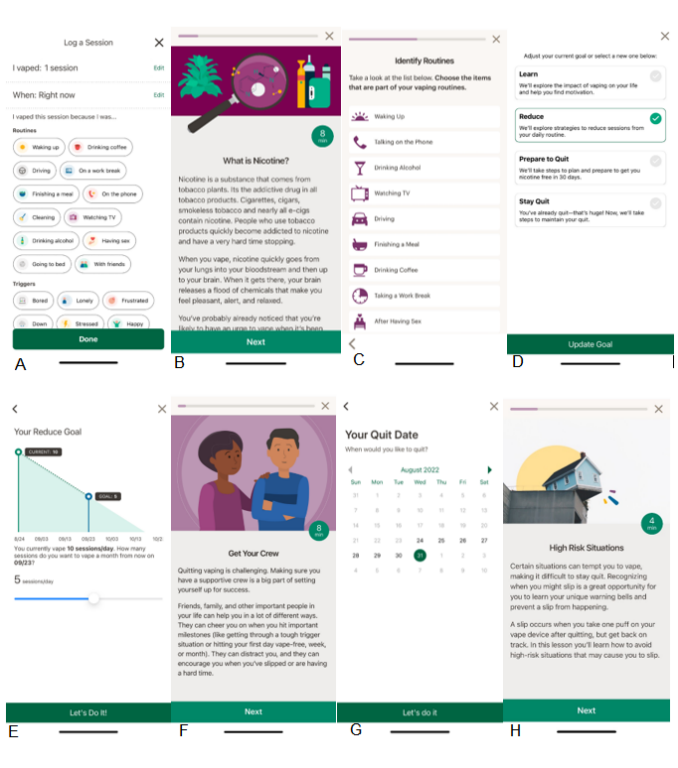
Representative screenshots from the Pivot vaping cessation app, which includes 4 pathways: Learn, Reduce, Prepare to Quit, and Stay Quit. (A) Logging a vape session (all pathways); (B) and (C) learning about nicotine and identifying routines related to vaping (Learn pathway); (D) selecting your goal (all pathways); (E) setting a reduction goal (Reduce pathway); (F) and (G) establishing a support network and setting a quit date (Prepare to Quit pathway); (H) learning about high-risk situations (Stay Quit pathway).

The Pivot app includes educational content and the ability to log vaping and track use over time, set a quit date, create a quit plan, play educational games, watch educational videos, interact with a dedicated human coach, and access the moderated web-based Pivot community discussion forum. The educational journey in the Pivot app comprises 4 pathways—Learn, Reduce, Prepare to Quit, and Stay Quit—and is designed to accommodate people along the spectrum of readiness to quit. Participants may choose to focus on building self-awareness and learn more about their vaping behavior, create and practice their plan to quit or reduce vaping, make a quit attempt, focus on staying quit, or any combination thereof. Accordingly, participants may navigate between pathways as desired to access content most relevant to their goals and needs. Figure 1 provides representative screenshots of the different Pivot pathways and functions.

Pivot users are assigned a human coach with whom they work one-on-one over the duration of Pivot (up to 12 months). Communication between the coach and the Pivot user is via asynchronous in-app messaging. Pivot coaches have National Board for Health and Wellness Coaching certification [32] and are certified tobacco treatment specialists. Coaches complete a tobacco treatment specialist training program accredited by the Council for Tobacco Treatment Training Programs [33].

Coaching is based on cognitive behavioral therapy and acceptance and commitment therapy and uses evidence-based techniques in tobacco cessation, such as motivational interviewing, with the aim of helping participants achieve their goals related to tobacco use. Coaching quality assurance is performed via an audit of 10% of all active coach-participant interactions every 12 weeks. The audit focuses on 6 specific tobacco treatment standards: building a personal plan to quit, addressing routines and triggers for vape use, addressing strategies for coping, addressing participant sources of support, demonstration of coach application of cognitive behavioral therapy and acceptance and commitment therapy to explore thoughts and feelings related to vape use and behavior change, and addressing a plan for avoiding relapse and managing slips. Participants may reach out to their coach whenever and however often they like. The coach reaches out periodically, approximately once per week, during the participant’s active use of Pivot.

Finally, Pivot users may access the moderated web-based discussion community through the Pivot app. The forum is moderated by a certified tobacco treatment specialist. The web-based community forum is a place to give and receive support and advice from others going through the Pivot program.

### Procedure

Participants were enrolled from January 2023 to June 2023. Participants were considered enrolled after completing the following 3 tasks: electronically signing the informed consent form, completing the baseline questionnaire, and completing registration on the Pivot app. On the basis of experience with the Pivot program for cigarette smoking cessation [10,11,13], it was estimated that approximately the first 6 months of study participation would primarily focus on active program use and the remaining 6 months in the study would focus on passive periodic follow-up. That said, participants had 12 months of full program access to use as they chose. This paper details outcomes from the first 6 months of the study.

### Data Collection

Data were collected electronically through participant input in the Pivot web-based registration form, Pivot app, and web-based questionnaires administered through SurveyMonkey (SurveyMonkey Inc). Data collection via web-based questionnaires was performed at baseline, every 2 weeks for the first 12 weeks, and at the 26-week follow-up. We collected data from participants every 2 weeks for the first 12 weeks to keep study questionnaires short enough to minimize participant inconvenience and, thereby, increase likelihood of questionnaire completion. Collection of data more closely related to program use, such as program acceptability and participant feedback, was primarily performed over the first 12 weeks of the study to obtain input temporally closest to program use. The 26-week follow-up was chosen expecting that most participants would be done using the program at this time based on previous experience with the Pivot smoking cessation program [10,11,13]. In addition, 26 weeks is comparable to follow-up time points of studies on digital interventions for vaping cessation [8,9].

For engagement, data were collected through the Pivot app to capture weeks active in the program, number and average duration of Pivot app sessions, most popular program features, and messages to and from the coach. For changes in attitudes toward quitting vaping, changes in vaping behavior, and participant feedback on the program, data were collected through web-based questionnaires.

### Outcome Variables and Measurement

#### Baseline Characteristics

Baseline characteristics included demographic information (age, gender, race, ethnicity, household income, educational level, employment status, and smartphone type), past cigarette use history if applicable (duration of cigarette smoking and methods used to quit smoking), vape use data (VSPD, type of vape device used, amount of nicotine used per week in milligrams, nicotine strength of e-liquid in milligrams per milliliter, duration of vaping, number of quit attempts over the previous 12 months, and methods used in previous quit attempts), and current use of other tobacco products. Dependence was assessed using the Penn State Electronic Cigarette Dependence Index (PSECDI) [34], along with symptoms of clinical depression using the Center for Epidemiologic Studies Depression Scale [35,36] and alcohol use through the Alcohol Use Disorders Identification Test–Concise [37].

#### Retention and Engagement

Several metrics of retention and engagement were used, including weeks active in the program, number of Pivot app sessions, number of weeks with at least one message from the participant to their coach, the number of messages to and from the coach, and the most frequently used program features. To be considered active in the program for the week in question, participants had to have at least one app session. An app session was defined as starting when a user performed any event and ending when the user was inactive for 30 minutes. This definition is from Mixpanel, which is a real-time user analytics platform that uses a database to store and analyze Pivot app use data. This definition is Mixpanel’s default definition of a session and, thus, broadly applied [38].

#### Attitudes Toward Quitting Vaping

Measurements assessing attitudes toward quitting vaping included desire to quit vaping (yes or no), anticipated success in quitting, and anticipated difficulty in staying quit. Anticipated success in quitting was assessed using the following question: “If you were to quit vaping/e-cigs right now, how successful would you be? 1 = Not at all successful, 10 = Completely successful.” Anticipated difficulty staying quit was assessed using the following question: “If you were to quit vaping/e-cigs right now, how difficult do you think it would be to stay vape/e-cig free? 1 = Really difficult to stay vape/e-cig free, 10 = Really easy to stay vape/e-cig free.”

#### Vaping Behavior

Several metrics assessed changes in vaping behavior: the PSECDI, quit attempts, 7- and 30-day PPA, continuous abstinence, and abstinence from all tobacco products. The PSECDI was evaluated in all participants at baseline and in the subset of participants who did not achieve 7-day PPA at 26 weeks. A quit attempt was defined as going at least 1 day without vaping even a single puff. Participants were considered to have achieved 7-day (30-day) PPA if they answered “no” to the following question: “In the last 7 (30) days have you vaped/used e-cigs, even a single puff?” Continuous abstinence was defined as self-report of 7-day (or greater) PPA at 12 weeks, self-report of 30-day PPA at 26 weeks, and 0 vaping sessions since 12 weeks. All changes in vaping behavior were self-reported.

#### Participant Feedback

Participants were asked to rate the ease of getting started in the Pivot program, how using the program affected their motivation to quit vaping, how program use affected the amount that they vaped each day, and how helpful the program is in helping someone quit vaping. Participants who reported 7-day PPA at 26 weeks were also asked how their ability to focus on daily tasks changed since quitting vaping.

### Sample Size

This is an exploratory study that will inform future research and Pivot program improvements. Review of the literature for similarly designed studies assessing digital interventions for vaping cessation yielded study sample sizes of 24 to 51 participants [6,7,9]. Accordingly, we aimed to enroll 70 to 100 participants, anticipating a 15% to 20% attrition rate based on previous studies [10-12,15], with the goal that at least 60 participants would complete the 26-week questionnaire.

### Statistical Analyses

Statistical analyses were conducted using all available data. For results in which a change from baseline could be measured, each participant’s baseline data served as their control to calculate a difference with a later time point.

For numerical data, we calculated the mean change from baseline and used a 1-sample, 2-tailed *t* test. Analyses were conducted using SAS (version 9.4; SAS Institute). Statistical significance was set at *P*<.05.

In the assessment of cessation (PPA), 2 sets of analyses were performed. In the first one, an ITT analysis, individuals who did not respond to PPA questions were assumed to be vaping. In addition, a study completer analysis was also performed, which only included individuals who completed the 26-week questionnaire.

We performed exploratory post hoc analyses using univariate linear regression to explore associations between baseline characteristics and the following: participant ratings of the ease or difficulty of program setup reported at 2 weeks, the number of study questionnaires completed, the number of app sessions completed, and the number of messages sent to the coach at 26 weeks. We evaluated each independent baseline variable as a predictor in a separate model using linear regression. We then performed multivariate stepwise linear regression including all independent variables that had a level of significance of *P*<.05 in the univariate regressions. Stepwise regression was performed using a significance level of *P*<.05 to enter and remain in the model.

For inclusion in the initial univariate regression analysis, eligible participant data had to be sought from the entire study cohort. For example, baseline questions asked only of former smokers (53/73, 73%) were not included. In addition, variables with multiple selections (eg, participants were asked to select all that applied) were converted to binary variables. For example, when asked to identify the reasons why they wanted to stop vaping, participants were presented with 4 options and could select all that applied. In this instance, the selection of “Health” (or any of the other possible response options) was converted to “yes” or “no.” Variables with less than 10% of responses in a category were not included as an arbitrary imbalance cutoff. For example, the gender category of “prefer not to state,” which was selected by 2 participants (2/73, 3%), was not included. Finally, certain categorical baseline variables were collapsed if possible to either simplify the analysis or meet the aforementioned 10% criteria. Analyses were conducted using SAS (version 9.4). *P* values of <.05 were considered statistically significant.

## Results

### Enrollment

From January 2023 to June 2023, a total of 598 web-based screening forms were received. Most did not advance further in the enrollment process, with the 3 following reasons being the most common: responses to the web-based screening questions rendered the individual ineligible (211/598, 35.3%), the potential participant could not be reached (71/598, 11.9%), and further contact was not attempted by study staff due to fulfillment of nonproportional quota sampling goals (64/598, 10.7%).

**Figure 2:**
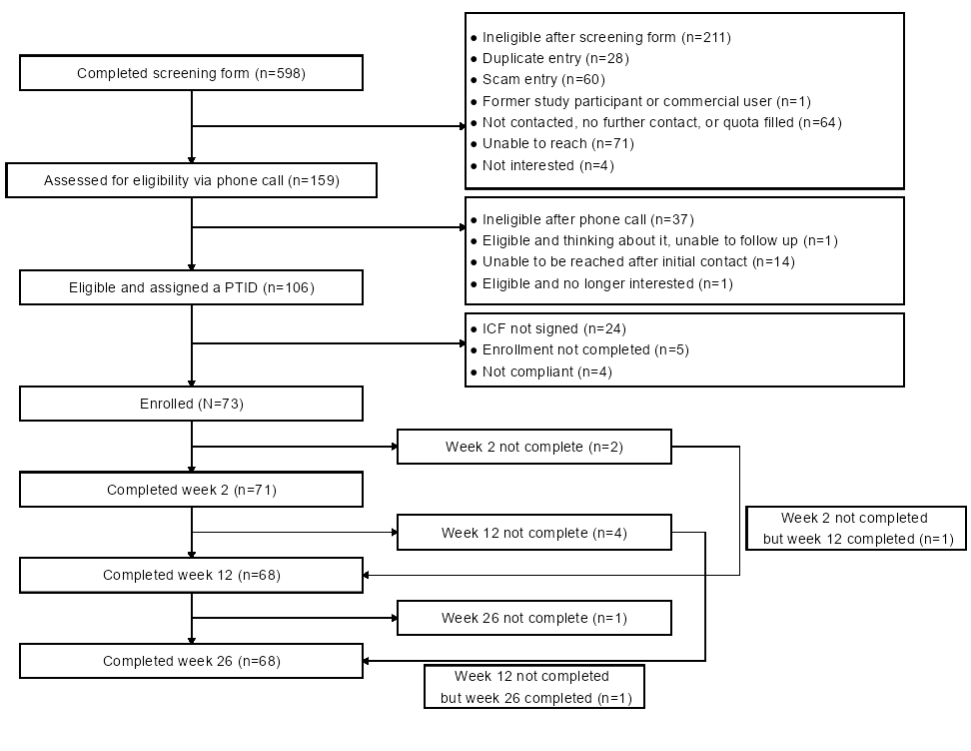
Study participant flow—CONSORT (Consolidated Standards of Reporting Trials) diagram. Participants in this remote, exploratory study were adults in the United States who vaped nicotine daily; they were recruited through web media from January 2023 to June 2023. ICF: informed consent form; PTID: participant identification number.

Study eligibility was assessed via a phone call in 63.1% (159/252) of potential participants. After the phone calls, electronic Health Insurance Portability and Accountability Act forms and informed consent forms were emailed to 66.7% (106/159) of these potential participants. Upon completion of these forms, registration links were emailed; 68.9% (73/106) of the participants completed the enrollment process. These 73 individuals comprise the ITT sample. Moreover, 93% (68/73) completed the 12- and 26-week questionnaires and comprised the study completer sample. Over the 26 weeks of the study, a total of 7% (5/73) of the participants were lost to follow-up. Study enrollment and attrition are shown in the participant flow diagram in Figure 2.

### Baseline Characteristics

The study sample consisted of 52% (38/73) women and 79% (58/73) participants who identified as White individuals, had a mean age of 37.4 (SD 11.3) years, vaped a mean of 14.6 (SD 6) VSPD, had a mean PSECDI of 13.9 (SD 3.1), and had been vaping for an average of 3.5 (SD 3) years. Most participants (53/73, 73%) were former smokers; these individuals had smoked for an average of 13.2 (SD 10.3) years. On average, participants had made 1.7 (SD 2.0) vaping quit attempts over the previous 12 months. While most nonproportional quota sampling goals were met, we had challenges enrolling people who had never been cigarette smokers (goal enrollment proportion: 40%-60%; actual enrollment proportion: 20/73, 27%) and people who did not identify as White individuals (goal enrollment proportion: >29%; actual enrollment proportion: 15/73, 21%). Key study demographic details are provided in Table 1; all baseline demographic details are provided in Multimedia Appendix 1.

**Table 1:** Baseline demographics; participants in this remote, exploratory cohort study were adults in the United States who vaped nicotine daily (N=73).

Characteristic				Values
**Demographics**				
	Age (y), mean (SD)			37.4 (11.3)
	**Gender, n (%)**			
		Women	38 (52)	
		Men	33 (45)	
		Prefer to self-describe	2 (3)	
	**Race, n (%)**			
		American Indian or Alaska native	1 (1)	
		Asian	3 (4)	
		Black or African American	4 (5)	
		Hispanic, Latino or Latina, or Spanish origin	4 (5)	
		Native Hawaiian or other Pacific Islander	1 (1)	
		White	58 (79)	
		Some other race	2 (3)	
	**Ethnicity, n (%)**			
		Hispanic, Latino or Latina, or Spanish origin	8 (11)	
		Not of Hispanic, Latino or Latina, or Spanish origin	65 (89)	
	**Educational level, n (%)**			
		High school or GED^a^	4 (5)	
		Some college	22 (30)	
		Associate (2-y) degree	9 (12)	
		Bachelor’s (4-y) degree	26 (36)	
		Master’s degree	10 (14)	
		Professional or doctorate degree	2 (3)	
	**Income (US $), n (%)**			
		<25,000	8 (11)	
		25,000-34,999	3 (4)	
		35,000-49,999	10 (14)	
		50,000-74,999	12 (16)	
		75,000-99,999	14 (19)	
		100,000-149,999	15 (21)	
		≥150,000	11 (15)	
	**Employment, n (%)**			
		Yes, ≥20 h/wk	54 (74)	
		Yes, <20 h/wk	7 (10)	
		No	12 (16)	
	**Smartphone, n (%)**			
		iPhone	43 (59)	
		Android	30 (41)	
**Vaping and quitting behavior**				
	Vape sessions per day^b^, mean (SD)			14.6 (6)
	Years vaping, mean (SD)			3.5 (3)
	Age when they first started thinking of themselves as someone who vapes (y), mean (SD)			33.9 (12)
	**Vape device used, n (%)**			
		Refillable	20 (27)	
		Prefilled or disposable (single use)	44 (60)	
		Both	9 (12)	
	Uses vape or e-cigarette flavors, n (%)			59 (81)
	**First vape session after waking, n (%)**			
		Within 5 min	40 (55)	
		6-15 min	18 (25)	
		16-30 min	9 (12)	
		31-60 min	3 (4)	
		61-120 min	2 (3)	
		After 120 min	1 (1)	
	**Vaping dependence via the PSECDI** ^c^ **, n (%)**			
		Low dependence	3 (4)	
		Medium dependence	20 (27)	
		High dependence	50 (68)	
	Quit attempts in the previous 12 months, mean (SD)			1.7 (2.0)
	**Attitudes toward quitting vaping, mean (SD)**			
		DTQ^d^	4.0 (2.8)	
		STQ^e^	4.9 (2.9)	
	Alcohol use behavior via the AUDIT-C^f^, mean (SD)			2.3 (2.3)
	Presence of depressive symptoms via the CES-D^g^, mean (SD)			9.8 (6.4)
**Current product and medication use, n (%)**				
	**Tobacco products used**			
		e-Cigarettes or vaping only	72 (99)	
		e-Cigarettes or vaping+hookah	1 (1)	
	**Other vape juice or e-liquid products used** ^h^			
		None	60 (82)	
		CBD^i^	5 (7)	
		THC^j^	11 (15)	
		Other	1 (1)	
	**Medications currently taking**			
		None	67 (92)	
		Bupropion (Zyban/Wellbutrin)	4 (5)	
		NRT^k^—gum	1 (1)	
		NRT—patch	1 (1)	
**Former smoking-related measures**				
	Former smokers, n (%)			53 (73)
	Years smoked, mean (SD)			13.2 (10.3)
	Started vaping to quit smoking, n (%)			43 (59)
	Continued vaping to quit smoking or stay quit, n (%)			30 (41)

^a^GED: General Educational Development.

^b^Participants were provided with a drop-down menu for vape sessions per day with a selection from 0 to >20; >20 was converted to 21.

^c^PSECDI: Penn State Electronic Cigarette Dependence Index; 10 items (where a score of 0-3 indicates no dependence, a score of 4-8 indicates low independence, a score of 9-12 indicates medium dependence, and a score of ≥13 indicates high dependence).

^d^DTQ: difficulty staying quit; “If you were to quit vaping/e-cigs right now, how difficult do you think it would be to stay vape free? (1=Really difficult to stay vape/e-cig free; 10=Really easy to stay vape/e-cig).”

^e^STQ: success in quitting; “If you were to quit vaping/e-cigs right now, how successful would you be? (1=not at all successful; 10=completely successful).”

^f^AUDIT-C: Alcohol Use Disorders Identification Test–Concise screening questionnaire; 3 items (where a score of 0 indicates no alcohol use and a score of ≥4 among men and ≥3 among women is considered positive for alcohol use disorder).

^g^CES-D: Center for Epidemiologic Studies Depression Scale; 10 items (where a higher score indicates greater symptoms of depression; a cutoff score of ≥10 indicates the presence of significant depressive symptoms).

^h^Participants were asked to select all that applied.

^i^CBD: cannabidiol.

^j^THC: tetrahydrocannabinol.

^k^NRT: nicotine replacement therapy.

### Retention and Engagement

**Figure 3:**
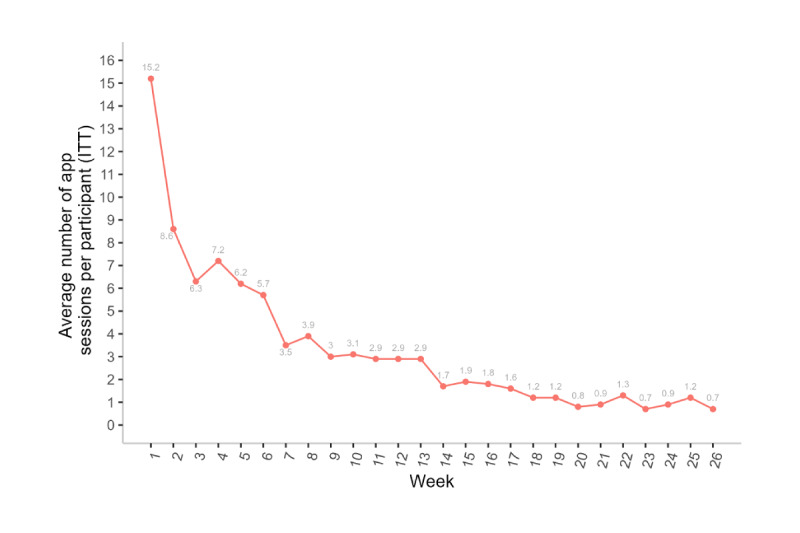
Average number of Pivot app sessions per participant by week (N=73). An app session was defined as starting when a user performed any event on the app and ending when the user was inactive for 30 minutes. ITT: intention to treat.

On average, participants were active in the program for 13.8 (SD 7.3) weeks. Of the 6372 total app sessions (from all participants combined) that occurred over 26 weeks, 3587 (56.3%) occurred during weeks 1 to 6, a total of 1414 (22.19%) occurred during weeks 7 to 12, a total of 812 (12.74%) occurred during weeks 13 to 18, and 559 (8.77%) occurred during weeks 19 to 26. Participants had an average of 87.3 (SD 99.9) sessions in the Pivot app. Figure 3 shows the average number of app sessions per participant by week; these values decreased over time from 15.2 (SD 16.2) sessions at week 1, an average of 7.2 (SD 9.6) at week 4, an average of 3.9 (SD 5.2) at week 8, an average of 2.9 (SD 5.6) at week 12, and an average of 0.7 (SD 1.8) at week 26.

**Figure 4:**
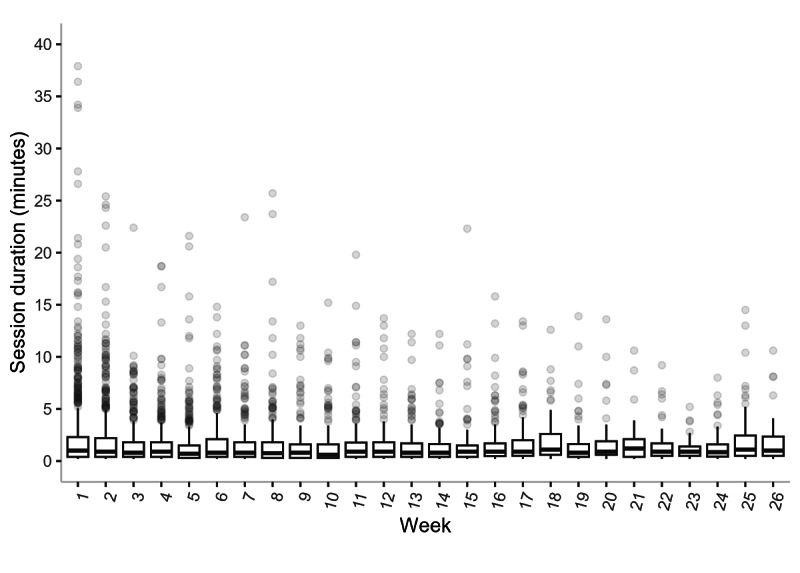
Average duration of Pivot app sessions (in minutes) by week among study participants (N=73). The horizontal line through each box represents the median, and the vertical length of the box along the y-axis represents the IQR (25%-75%).

Figure 4 details the average duration of app sessions by week. The median app session duration remained consistent over 26 weeks at approximately 1 minute.

The most popular program features were the web-based community forum and messaging one’s coach; 95% (69/73) of the participants accessed these features over 26 weeks.

**Figure 5:**
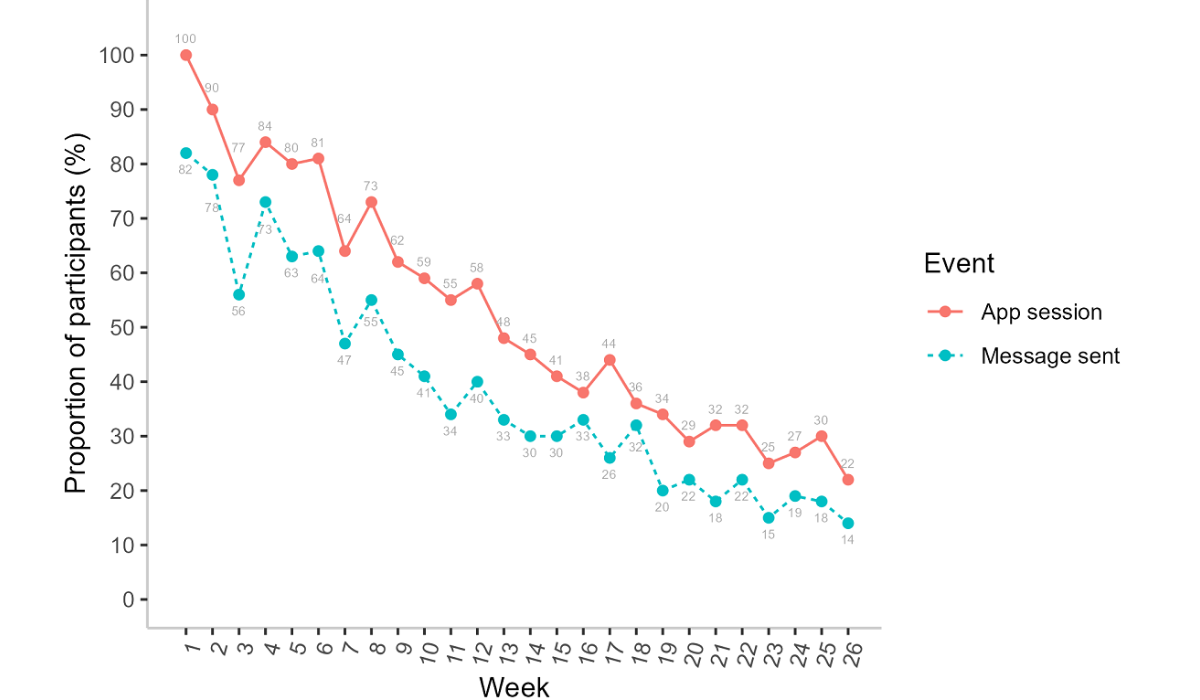
Proportion of participants with at least one Pivot app event by week (intention to treat; N=73). Messages sent by participants to the coach are a subset of app events.

Participants had an average of 10.1 (SD 6.8) weeks in which they sent at least one message to their coach. Figure 5 shows the proportion of participants who sent at least one message to their coach by study week, which was 82% (60/73) at week 1, a total of 55% (40/73) at week 8, a total of 40% (29/73) at week 12, and 14% (10/73) at week 26.

**Figure 6:**
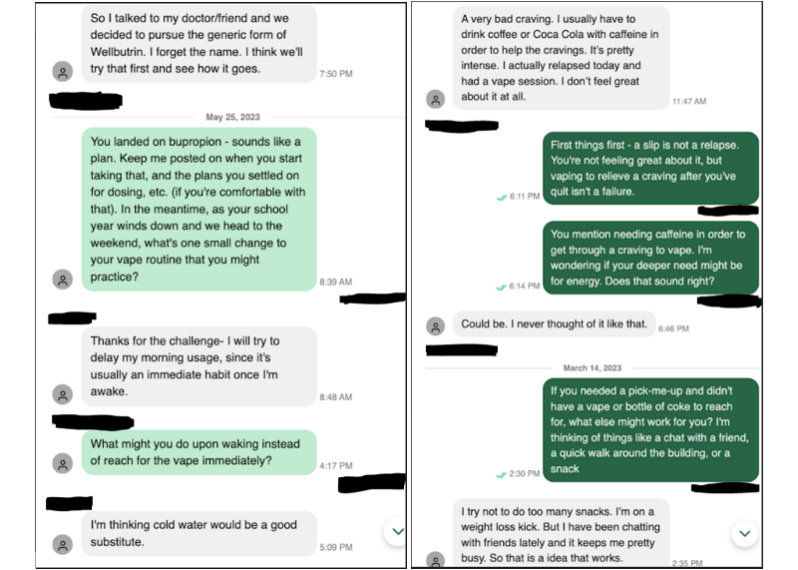
Examples of coach and study participant message interactions from 2 different participants. In the Pivot vaping cessation program, communication between the coach and the Pivot user took place via asynchronous in-app messaging.

Overall, of the 7471 messages sent during weeks 1 to 26, a total of 4727 (63.27%) were from the coach to the participant, and 2744 (36.73%) were from the participant to the coach. Over the 26-week study period, the mean total number of messages sent to the coach from the participant was 37.6 (SD 42.3) per participant. The mean total number of messages sent to the participant from the coach was 64.7 (SD 53.7) per participant. A total of 5% (4/73) of the participants never sent a message to their coach during the study. The asynchronous nature of coaching presents a challenge in categorizing a participant message as sent in response to coach outreach versus a proactive self-initiated outreach. One might consider the time between messages as an indirect indicator, with messages from the participant to the coach sent further out from the most recent previous message being more likely to be proactive and self-initiated by the participant. Of the 2744 messages sent from the participant to the coach, 797 (29.05%) were sent >24 hours after the most recent previous message, 213 (7.76%) were sent between 6 and 24 hours after the most recent previous message, and 1734 (63.19%) were sent from <5 minutes to 5 hours 59 minutes after the most recent previous message. Figure 6 provides examples of coach and participant message interactions.

### Attitudes Toward Quitting Vaping

When asked at 4 weeks, all respondents (70/70, 100%) indicated that they wanted to completely stop vaping. From baseline to 12 weeks, participants reported substantial improvement in their confidence to quit vaping and maintain their quit status (Table 2).

**Table 2:** Changes in attitudes toward quitting vaping among study participants (adult daily vapers in the United States) from baseline to 12 weeks after enrollment (N=73).

Measure		Participants, n (%)	Score, mean (SD)	*P* value^a^
**STQ** ^b^				<.001
	Baseline	73 (100)	4.9 (2.9)	
	12 weeks	68 (93)	7.0 (3.0)	
	Change	—^c^	2.0 (3.2)	
**DTQ** ^d^				<.001
	Baseline	73 (100)	4.0 (2.8)	
	12 weeks	68 (93)	6.2 (3.1)	
	Change	—	2.0 (3.9)	

^a^Paired 2-tailed *t* test change (ie, difference from baseline to 12 weeks).

^b^STQ: success in quitting; “If you were to quit vaping/e-cigs right now, how successful would you be? 1=not at all successful, 10=completely successful.”

^c^Not applicable.

^d^DTQ: difficulty in staying quit; “If you were to quit vaping/e-cigs right now, how difficult do you think it would be to stay vape/e-cig free? 1 = Really difficult to stay vape/e-cig free, 10 = Really easy to stay vape/e-cig free.”

### Vaping Behavior

Most participants (64/73, 88%) made at least one quit attempt during the study. Vaping cessation rates are shown in Table 3, with almost half (33/73, 45%) of the participants achieving 30-day PPA at 26 weeks. All participants who reported 7-day PPA at 26 weeks also indicated that they were not using any other tobacco products.

Among participants who did not report 7-day PPA at 26 weeks (33/73, 45%), a reduction was observed on the PSECDI from baseline (mean 13.9, SD 3.1) to 26 weeks (mean 10.8, SD 4.5), with a significant change (mean –3.2, SD 3.9; *P*<.001). Of these 33 participants, almost half (n=16, 48%) improved their electronic cigarette dependence category at 26 weeks (Table 4).

**Table 3:** Vaping cessation rates among study participants (adult daily vapers in the United States) at 12 and 26 weeks after enrollment (N=73).

Outcome		Participants, n (%)
**7-day PPA** ^a^		
	12-week ITT^b^	28 (38)
	26-week ITT^c^	35 (48)
	12-week responder analysis^d^	28 (41)
	26-week responder analysis^e^	35 (51)
**30-day PPA** ^f^		
	12-week ITT^b^	22 (30)
	26-week ITT^c^	33 (45)
	12-week responder analysis^d^	22 (32)
	26-week responder analysis^e^	33 (49)
**Self-reported continuous abstinence** ^g^		
	26-week ITT^c^	22 (30)
	26-week responder analysis^h^	22 (33)
**Abstinence from all tobacco products**		
	26-week ITT^c^	35 (48)
	26-week responder analysis^e^	35 (51)

^a^PPA: point-prevalence abstinence; “In the last 7 days have you vaped/used e-cigs, even a single puff? Select one. Yes or No.”

^b^ITT: intention-to-treat analysis; N=73.

^c^N=73.

^d^N=68.

^e^N=68.

^f^“In the last 30 days have you vaped/used e-cigs, even a single puff? Select one. Yes or No.”

^g^Self-report of 7-day (or greater) PPA at 12 weeks, self-report of 30-day PPA at 26 weeks, and 0 vape sessions since 12 weeks.

^h^Responder analysis for continuous abstinence at 26 weeks; N=67 (participants who responded to both the 12- and 26-week questionnaires).

**Table 4:** Change in electronic cigarette dependence from baseline to 26 weeks among study participants (adult daily vapers in the United States) who did not report 7-day point-prevalence abstinence at 26 weeks (N=33).

PSECDI^a^ (baseline)	PSECDI (26 weeks), n (%)				
	Not dependent	Low dependence	Medium dependence	High dependence	Sum
Not dependent	0 (0)	0 (0)	0 (0)	0 (0)	0 (0)
Low dependence	0 (0)^b^	1 (3)^c^	0 (0)	0 (0)	1 (3)
Medium dependence	1 (3)^b^	5 (15)^b^	3 (9)^c^	2 (6)^d^	11 (33)
High dependence	1 (3)^b^	1 (3)^b^	8 (24)^b^	11 (33)^c^	21 (64)
Sum	2 (6)	7 (21)	11 (33)	13 (39)	33 (100)

^a^PSECDI: Penn State Electronic Cigarette Dependence Index; 10 items (where a score of 0-3 indicates no dependence, a score of 4-8 indicates low independence, a score of 9-12 indicates medium dependence, and a score of ≥13 indicates high dependence).

^b^Participant PSECDI improved from baseline to 26 weeks.

^c^Participant PSECDI did not change from baseline to 26 weeks.

^d^Participant PSECDI worsened from baseline to 26 weeks.

### Participant Feedback

On a scale from 1 to 10 (1=very difficult; 10=very easy), participants rated the ease of getting started in the Pivot program with a mean of 8.3 (SD 2.3). When asked at 2 weeks, 72% (51/71) of the respondents reported that using Pivot increased their motivation to quit vaping; the remainder (20/71, 28%) indicated that using Pivot did not affect their motivation to quit. When asked at 4 weeks, 79% (55/70) of the respondents reported that using the Pivot program decreased the amount they vaped per day; the remainder (15/70, 21%) responded that using Pivot did not affect the amount they vaped per day. When asked at 12 weeks about Pivot’s ability to help someone quit vaping, the response options were extremely helpful to quit vaping (15/68, 22%), helpful to quit vaping (40/68, 59%), did not affect being able to quit vaping (13/68, 19%), or made quitting vaping more difficult (0%).

Most participants who reported 7-day PPA at 26 weeks reported that their ability to focus on daily tasks had increased since quitting vaping (24/35, 69%); 23% (8/35) reported no change, and 9% (3/35) reported a decreased ability to focus on daily tasks.

### Associations With Feedback on Program Setup, Completion of Study Questionnaires, and Program Use

In the final model that evaluated program setup, participants who were employed tended to rate it as being easier to use compared to unemployed individuals (1.7, 95% CI 0.4-3.0; *P*=.01). In contrast, baseline report of more quit attempts over the previous 12 months was associated with participants rating the program setup as more difficult (–0.4, 95% CI –0.6 to –0.1; *P*=.003).

Regarding the number of study questionnaires completed, the difficulty in quitting rating was the only statistically significant variable and had a positive association (0.2, 95% CI 0.01-0.3; *P*=.04); in other words, increasing number of completed study questionnaires was associated with increasing DTQ score (which reflected greater expected ease in staying quit).

There were 3 baseline characteristics associated with a greater number of app sessions over the 26 weeks. These were having a 2-year college degree or higher (46.1, 95% CI 6.6-85.5; *P*=.02) compared to no college education, having started vaping to manage stress (49.9, 95% CI 7.3-92.4; *P*=.02) versus not, and having more close friends who smoke cigarettes (12.8, 95% CI 6.6-85.5; *P*=.02).

There were 2 characteristics positively associated and 2 characteristics negatively associated with the number of messages that a participant sent to their coach. Having a 2-year college degree or higher (21.9, 95% CI 3.7-40.2; *P*=.02) compared to not and having started vaping due to boredom (39.8, 95% CI 14.1-65.5; *P*=.002) versus not were both associated with a greater number of sent messages. In contrast, individuals who earned <US $50,000 (–33.5, 95% CI –43.9 to –0.9; *P*=.04) and those who earned >US $100,000 (–22.4, 95% CI –53.6 to –13.6; *P*=.001) sent fewer messages to their coach compared to participants who earned between US $50,000 and US $99,999.

The analysis is detailed in Multimedia Appendix 2. Due to the small sample size and post hoc nature of this analysis, we encourage caution with interpretation and think of these data primarily as interesting to consider in the design of future studies.

## Discussion

### Principal Findings

Pivot is a well-established digital tobacco cessation program with historical focus on smoking cessation [10-15]. This exploratory study is the first to assess the Pivot vaping cessation program in American adults who vape daily. In this study, 93% (68/73) of the participants completed the 12- and 26-week questionnaires. On average, participants were active in Pivot for 13.8 (SD 7.3) weeks, had 87.3 (SD 99.9) app sessions, and sent 37.6 (SD 42.3) messages to their coach over 26 weeks. The most commonly used program features were messaging one’s coach and accessing the moderated web-based community forum. Many participants (51/71, 72%) reported increased motivation to quit vaping. Participants had significant improvements in their confidence to quit vaping and confidence to stay quit. Regarding early indicators of cessation rates, 45% (33/73; ITT) of the participants achieved 30-day PPA at 26 weeks, and 30% (22/73; ITT) achieved continuous abstinence by 12 weeks that lasted up to 26 weeks.

The uptick in vaping over the last several years begets a need for evidence-based cessation resources such as the Pivot program; >60% of American adults who vape are interested in quitting [5]. This need is further underscored by the evolving role of vaping in cigarette smoking cessation [39]. Notably, 59% (43/73) of the participants in this study started vaping to help them quit smoking; the next step for them is to quit vaping. Accordingly, investigation of vaping cessation methods and interventions such as the one in this study is timely and salient.

### Comparison With Prior Work

Digital intervention for vaping cessation is a nascent but growing field. The limited published evidence on such programs is further impacted by differences in study designs and interventions. Webb et al [6] assessed similar engagement outcomes to those in this study in their evaluation of the Quit Genius-Vaping tobacco cessation program in 51 adults. They reported that participants opened the Quit Genius-Vaping app an average of 25.2 (SD 31.8) times, sent 7.3 (SD 8.6) messages to their coach, and received 12.4 (SD 8.2) messages from their coach. These values are all lower than those reported in this study for comparable outcomes; however, it is important to note that the study by Webb et al [6] was shorter in duration, with outcomes collected 1 month after the target quit date and the target quit date occurring an average of 8 days after app download. In both studies, coaching was highly regarded by participants.

Published 30-day PPA rates from digital interventions range from 24% to 100% for time points ranging from approximately 3 weeks to 7 months [6-9,40]. Importantly, most of these rates are from small studies (N=8-51). The notable exception is the RCT by Graham et al [8] (N=2588); at the 7-month follow-up, self-reported 30-day PPA rates were 24.08% (314/1304; 95% CI 21.8%-26.5%) in intervention participants, who received an automated, tailored, interactive SMS text messaging program (This is Quitting), versus 18.61% (239/1284; 95% CI 16.7%-20.8%) in control participants, who received assessment-only (odds ratio 1.39, 95% CI 1.15-1.68; *P*<.001). The 30-day PPA in 45% (33/73; ITT) of the participants at 6 months in this study sits favorably within this published range of outcomes. That said, the ability to compare vaping cessation outcomes among these studies is significantly limited by differences in study design, particularly sample size and whether a study had a randomized design. Overall, it is important to acknowledge that these and most of the previously published cessation data provide an early signal of vaping cessation outcomes that requires further bearing out via rigorous comparative study.

Shifting consideration more broadly to vaping cessation in general, there are several reviews (2 systematic reviews and 1 scoping review) inclusive of studies evaluating digital, pharmacological, and behavioral interventions and no interventions at all [41-43]. These reviews, published from 2022 to 2023, include between 12 and 79 articles each and vary in article inclusion and exclusion criteria but broadly focus on published studies addressing vaping cessation. Review of these systematic reviews reveals a few recurring themes—(1) consensus that there is a paucity of data on vaping cessation interventions with an associated gap in evidence-based clinical care; (2) the need for rigorous assessment of several aspects of vaping cessation, including established tools used for tobacco dependence that have been tailored to e-cigarette users, such as behavioral counseling and pharmacotherapy, and among certain populations, such as former smokers who started vaping to help them quit cigarettes, adults, and minority groups; (3) the fact that adults (aged ≥25 y) are particularly underrepresented in an already limited dataset and will possibly require different intervention methods than youth and adolescents; and (4) early data on mobile interventions are encouraging and suggest a role for smartphone-based care delivery—to be borne out by future research.

### Strengths and Limitations

The strengths of this study include the length of follow-up of 26 weeks, high participant retention rate for data collection (study questionnaire completion was 68/73, 93% at 12 and 26 weeks), and inclusion of participants who were not ready to quit in the short term to reflect a diversity in cessation readiness.

This study has a few limitations. First, we did not achieve our nonproportional quota enrollment goals; we aimed for more representation of people who had never been cigarette smokers and people who did not identify as White individuals. The proportion of our participants who were former smokers (53/73, 73%) versus never smokers (20/73, 27%) was higher than that reported in the general population of current American adult e-cigarette users, in which former smokers are approximately 1.6 to 2.1 times as common as never smokers [5,16,17,44]. Regarding representation of people of different races, our enrollment of people identifying as White individuals (58/73, 79%) was slightly higher than that reported in the US general population of adults who vape regularly (63%-74%) [16-18,45]. Our experience is not unique; others have also reported challenges in recruiting for studies assessing digital tobacco cessation programs [20,46]. Our future recruitment efforts will be informed by these shortfalls with additional focus on these groups.

In addition, other aspects of study design may limit the generalizability of the results to the wider population of adults who vape daily. During screening, all participants indicated that they were seriously thinking of quitting vaping within the next 6 months and that they were interested in working with a vaping cessation coach; both may suggest an increased likelihood of quitting. We incorporated these screening criteria given that individuals with some degree of readiness to quit and interest in working with a coach are the most likely users of this program. While we do not have comparable data in the vaping population, it is interesting to note that, in a cohort study of Pivot for cigarette smoking cessation (N=319), individuals who indicated that they were not seriously thinking of quitting smoking in the next 30 days at baseline had comparable 4-month cessation outcomes to those of individuals who indicated that they were thinking of quitting [11].

It is also important to interpret the vaping cessation rates herein with caution given the lack of a control group and the sample of participants with some degree of readiness to quit and interest in working with a coach. Overall, this was an exploratory study to establish early outcomes. As such, the ability to draw conclusions about the effectiveness of Pivot for vaping cessation is limited. Logical and appropriate next steps include accordingly informed comparative investigation and assessment of cost-effectiveness, both necessary in the holistic assessment of a novel intervention.

In addition, participants were compensated for taking part; we cannot exclude some degree of associated influence on outcomes. We attempted to limit the influence of compensation as follows: not linking compensation to outcomes or program use, delaying payments 2 to 3 weeks after the completion of compensated events, and incorporation of fair but conservative payment amounts (US $25 to US $50 per completed study questionnaire for up to a total of US $250 for 8 questionnaires over 26 weeks).

Another limitation is the lack of biovalidation of cessation outcomes. Biovalidation in remote digital vaping cessation studies presents various challenges, including distinguishing between use of e-cigarettes and other sources of nicotine, cost, logistics, resource use, and participant compliance in sample collection [47,48]. Vaping and smoking cessation studies also demonstrate high concordance between self-reported and biovalidated outcomes [7,49-52]. Considering the existing data from vaping and smoking cessation studies along with the well-documented challenges of biovalidation in remote studies, we felt that self-reported outcomes were most appropriate for this initial assessment of Pivot for vaping cessation.

### Conclusions

As the prevalence of e-cigarette use rises, so too does the need for effective cessation interventions. The ubiquitous nature of smartphone ownership makes mobile programs such as Pivot increasingly attractive for individuals seeking to quit vaping and for the health care providers who would like to provide support but are faced with conflicting demands on their limited time and resources. The practical implications of this study, including consideration of participant engagement, change in attitudes and motivations regarding vaping cessation, early cessation outcomes, and feedback, suggest a role for Pivot in vaping cessation. Future comparative study and assessment of this and other digital interventions will further bear out optimal approaches to this growing public health problem.

## Appendix

Multimedia Appendix 1 Participant baseline data (complete).

Multimedia Appendix 2 Regression analysis.

## Abbreviations

ITT: intention to treat
NRT: nicotine replacement therapy
PPA: point-prevalence abstinence
PSECDI: Penn State Electronic Cigarette Dependence Index
RCT: randomized controlled trial
VSPD: vape sessions per day

## Data Availability

The data sets generated during and analyzed during this study are available from the corresponding author on reasonable request.
