# Supplementary material for: Outcomes of a Comprehensive Mobile Vaping Cessation Program in Adults Who Vape Daily: Cohort Study
Source: JMIR Form Res. 2024 Oct 28;8:e57376. doi: 10.2196/57376 (PMC11555445; doi:10.2196/57376)
Supplement: Multimedia Appendix 1 [file formative_v8i1e57376_app1.docx]

**Table 1.** Participant baseline data (N=73)

| **Characteristic** | | | **All**  **(N=73)** |
| --- | --- | --- | --- |
| **Demographics** | | |  |
|  | Age (years), mean (SD) | | 37.4 (11.3) |
|  | Gender, n (%) | |  |
|  |  | Woman | 38 (52) |
|  |  | Man | 33 (45) |
|  |  | Prefer to self-describe | 2 (3) |
|  | Race, n (%) | |  |
|  |  | American Indian or Alaska Native | 1 (1) |
|  |  | Asian | 3 (4) |
|  |  | Black or African American | 4 (5) |
|  |  | Hispanic, Latino/Latina, or Spanish Origin | 4 (5) |
|  |  | Native Hawaiian or Other Pacific Islander | 1 (1) |
|  |  | White | 58 (79) |
|  |  | Some other race | 2 (3) |
|  | Ethnicity, n (%) | |  |
|  |  | Hispanic, Latino/Latina, or Spanish Origin | 8 (11) |
|  |  | Not of Hispanic, Latino/Latina, or Spanish Origin | 65 (89) |
|  | Education, n (%) | |  |
|  |  | High school/GED | 4 (5) |
|  |  | Some college | 22 (30) |
|  |  | Associate’s (2-year) degree | 9 (12) |
|  |  | Bachelor’s (4-year) degree | 26 (36) |
|  |  | Master’s degree | 10 (14) |
|  |  | Professional or doctorate degree | 2 (3) |
|  | Income, n (%) | |  |
|  |  | Less than $25,000 | 8 (11) |
|  |  | $25,000 to $34,999 | 3 (4) |
|  |  | $35,000 to $49,999 | 10 (14) |
|  |  | $50,000 to $74,999 | 12 (16) |
|  |  | $75,000 to $99,999 | 14 (19) |
|  |  | $100,000 to $149,999 | 15 (21) |
|  |  | $150,000 or more | 11 (15) |
|  | Employment, n (%) | |  |
|  |  | Yes, 20 or more hours per week | 54 (74) |
|  |  | Yes, less than 20 hours per week | 7 (10) |
|  |  | No | 12 (16) |
|  | Self-reported health, n (%) | |  |
|  |  | Excellent | 11 (15) |
|  |  | Very Good | 37 (51) |
|  |  | Good | 17 (23) |
|  |  | Fair | 5 (7) |
|  |  | Poor | 3 (4) |
|  | Smartphone, n (%) | |  |
|  |  | iPhone | 43 (59) |
|  |  | Android | 30 (41) |
| **Vaping and Quitting Behavior** | | |  |
|  | Vape sessions per day^a^, mean (SD) | | 14.6 (6) |
|  | Years vaping, mean (SD) | | 3.5 (3) |
|  | Age first tried vaping, mean (SD) | | 32.2 (12) |
|  | Age first started thinking of self as someone who vapes, mean (SD) | | 33.9 (12) |
|  | Vape device used, n (%) | |  |
|  |  | Refillable | 20 (27) |
|  |  | Prefilled/ disposable (single use) | 44 (60) |
|  |  | Both | 9 (12) |
|  | Uses vape/e-cig flavor(s), n (%) | | 59 (81) |
|  | Nicotine concentration (mg/mL) used, mean (SD) | |  |
|  |  | Refillable | 22.9 (18) |
|  |  | Prefilled/ disposable (single use) | 35.6 (20) |
|  | Nicotine type used^b^, n (%) | |  |
|  |  | Freebase nicotine | 11 (15) |
|  |  | Nicotine salt | 46 (63) |
|  |  | I don’t know | 25 (34) |
|  | Amount of nicotine (mg) used per week, mean (SD) | |  |
|  |  | Refillable | 1803.5 (4095) |
|  |  | Prefilled/ disposable (single use) | 2312.4 (3913) |
|  | Vape juice/e-liquid volume of primary device, mean (SD) | |  |
|  |  | Refillable | 3.4 (2.9) |
|  |  | Prefilled/ disposable (single use) – cartridge volume | 6.8 (8.7) |
|  | First vape session after waking, n (%) | |  |
|  |  | Within 5 minutes | 40 (55) |
|  |  | 6 to 15 minutes | 18 (25) |
|  |  | 16 to 30 minutes | 9 (12) |
|  |  | 31 to 60 minutes | 3 (4) |
|  |  | 61 to 120 minutes | 2 (3) |
|  |  | After 120 minutes | 1 (1) |
|  | Vaping dependence via PSECDI^c^, n (%) | |  |
|  |  | Low dependence | 3 (4) |
|  |  | Medium dependence | 20 (27) |
|  |  | High dependence | 50 (69) |
|  | Reasons started vaping^d^, n (%) | |  |
|  |  | Boredom | 9 (12) |
|  |  | Curiosity | 23 (32) |
|  |  | Flavor choices | 12 (16) |
|  |  | Lack of odor | 12 (16) |
|  |  | My family members vape | 3 (4) |
|  |  | My friends/peer group vape | 24 (33) |
|  |  | Social image | 3 (4) |
|  |  | To help me quit smoking | 43 (59) |
|  |  | To manage stress | 21 (29) |
|  |  | Because I can vape/use e-cigs indoors | 18 (25) |
|  |  | Other | 3 (4) |
|  | Reasons continued to vape^d^, n (%) | |  |
|  |  | It was too difficult to quit | 45 (62) |
|  |  | Boredom | 11 (15) |
|  |  | Flavor choices | 15 (21) |
|  |  | Lack of odor | 10 (14) |
|  |  | My family members vape | 1 (1) |
|  |  | My friends/peer group vape | 12 (16) |
|  |  | Social image | 0 (0) |
|  |  | To help me quit smoking/stay quit | 30 (41) |
|  |  | To manage stress | 34 (47) |
|  |  | Because I can vape/use e-cigs indoors | 21 (29) |
|  |  | Other | 2 (3) |
|  | Quit attempts in the past 12 months, mean (SD) | | 1.7 (2.0) |
|  | Methods used in past vaping quit attempts^e^, n (%) | |  |
|  |  | Cold turkey | 39 (53) |
|  |  | Taper - decrease number of vape sessions per day, puffs per session, and/or the length (in time) of vape sessions | 34 (47) |
|  |  | Taper - decrease nicotine concentration in my vape juice/e-liquid | 25 (34) |
|  |  | Medication - Nicotine Replacement Therapy (NRT) | 8 (11) |
|  |  | Medication - Bupropion/Zyban/Wellbutrin | 2 (3) |
|  |  | Medication - Varenicline/Chantix | 3 (4) |
|  |  | Counseling - phone, group, in-person, text | 0 (0) |
|  |  | Class(es) (in-person or online) | 0 (0) |
|  |  | Smartphone app | 2 (3) |
|  |  | Hypnotherapy | 1 (1) |
|  |  | Acupuncture | 1 (1) |
|  |  | Other | 3 (4) |
|  |  | None | 19 (26) |
|  | Reasons to stop vaping^f^, mean (SD) | |  |
|  |  | Health | 1.4 (0.8) |
|  |  | Financial cost | 2.5 (1.0) |
|  |  | Freedom from addiction | 2.1 (0.7) |
|  |  | Social impacts (impacts on relationships, how you are perceived by others) | 3.3 (0.7) |
|  | Attitudes Towards Quitting Vaping | |  |
|  |  | DTQ^g^, mean (SD) | 4.0 (2.8) |
|  |  | STQ^h^, mean (SD) | 4.9 (2.9) |
|  | Please rate each issue as it relates to challenges you may face when quitting vaping/e-cigs^i^, mean (SD) | |  |
|  |  | Weight gain | 1.9 (1.3) |
|  |  | Expected anxiety and/or emotional discomfort | 3.5 (1.3) |
|  |  | Expected physical discomfort | 2.9 (1.2) |
|  |  | Changes in friend or family relationships | 2.0 (1.3) |
|  | How is vaping/using e-cigs and drinking alcohol linked for you, n (%) | |  |
|  |  | Not applicable, I don’t drink alcohol | 26 (36) |
|  |  | Not linked | 16 (22) |
|  |  | Linked, I vape/use e-cigs more when I drink alcohol | 26 (36) |
|  |  | Linked, I vape/use e-cigs less when I drink alcohol | 5 (7) |
|  | Alcohol use behavior via AUDIT-C^j^, mean (SD) | | 2.3 (2.3) |
|  | Presence of depressive symptoms via CES-D^k^, mean (SD) | | 9.8 (6.4) |
| **Current Product and Medication Use** | | |  |
|  | Tobacco products used, n (%) | |  |
|  |  | E-cigarettes/vaping only | 72 (99) |
|  |  | E-cigarettes/vaping + Hookah | 1 (1) |
|  | Other vape juice/e-liquid products used^e^, n (%) | |  |
|  |  | None | 60 (82) |
|  |  | CBD | 5 (7) |
|  |  | THC | 11 (15) |
|  |  | Other | 1 (1) |
|  | Medications currently taking, n (%) | |  |
|  |  | None | 67 (92) |
|  |  | Bupropion/Zyban/Wellbutrin | 4 (5) |
|  |  | Nicotine Replacement Therapy – gum | 1 (1) |
|  |  | Nicotine Replacement Therapy – patch | 1 (1) |
| **Former Smoking-Related Measures** | | |  |
|  | Former smoker, n (%) | | 53 (73) |
|  |  | Years smoked, mean (SD) | 13.2 (10.3) |
|  | Started vaping to quit smoking, n (%) | | 43 (59) |
|  | Continue vaping to quit smoking/ stay quit, n (%) | | 30 (41) |
|  | Methods used in past smoking quit attempts^e^, n (%) | |  |
|  |  | Cold turkey | 24 (45) |
|  |  | Medication - Nicotine Replacement Therapy (NRT) | 9 (17) |
|  |  | Medication - Bupropion/Zyban/Wellbutrin | 3 (6) |
|  |  | Medication - Varenicline/Chantix | 5 (9) |
|  |  | Counseling - phone, group, in-person, text | 1 (2) |
|  |  | E-cigarettes/vaping | 43 (81) |
|  |  | Class(es) (in-person or online) | 0 (0) |
|  |  | Smartphone app | 1 (2) |
|  |  | Hypnotherapy | 1 (2) |
|  |  | Acupuncture | 0 (0) |
|  | Please rate each issue as it relates to challenges you may face when quitting vaping/e-cigs^i^, mean (SD) | |  |
|  |  | Weight gain | 3.1 (1.7) |
|  |  | Expected anxiety and/or emotional discomfort | 4.0 (1.3) |
|  |  | Expected physical discomfort | 3.1 (1.4) |
|  |  | Changes in friend or family relationships | 1.9 (1.3) |
|  |  | Fear of relapsing to smoking cigarettes | 2.6 (1.5) |
| **Living and Social Environment** | | |  |
|  | Of the adults you live with, how many vape/use e-cigs, mean (SD) | | 0.3 (0.5) |
|  | Lives with a romantic partner who vapes/uses e-cigs, n (%) | | 15 (21) |
|  | Number of close friends who vape/use e-cigs, mean (SD) | | 3.2 (3.1) |
|  | Of the adults you live with, how many smoke cigarettes, mean (SD) | | 0.1 (0.4) |
|  | Lives with a romantic partner who smokes cigarettes, n (%) | | 5 (7) |
|  | Number of close friends who smoke cigarettes, mean (SD) | | 1.7 (2.2) |
|  | How much would quitting vaping positively influence others in your “online” world, scale 1-10, mean (SD) | | 3.9 (3.3) |
|  | How much would quitting vaping positively influence others in your “real” world, scale 1-10, mean (SD) | | 6.6 (3.4) |
| ^a^Participants were provided with a dropdown for vape sessions per day with a selection from 0 to >20. >20 was converted to 21.  ^b^Participants selected nicotine type for each device type they used; participants using both prefilled and refillable devices are counted twice.  ^c^PSECDI: Penn State Electronic Cigarette Dependence Index, 10 items (where a score of 0-3 indicates no dependence, a score of 4-8 indicates low independence, a score of 9-12 indicates medium dependence, and a score of 13+ indicates high dependence)  ^d^Participants were asked to select up to their top 3 answers.  ^e^Participants were asked to select all that apply.  ^f^Participants were asked to select all reasons why they would like to stop vaping/using e-cigs. Then they were asked to rank order their reasons to quit starting with a rank of 1 for the most important reason. (A lower average rank indicates greater importance).  ^g^DTQ difficulty to stay quit; If you were to quit vaping/e-cigs right now, how difficult do you think it would be to stay vape free? (1=Really difficult to stay vape/e-cig free; 10=Really easy to stay vape/e-cig)  ^h^STQ success to quit; If you were to quit vaping/e-cigs right now, how successful would you be? (1=not at all successful; 10=completely successful)  ^i^Participants were asked to rate each issue on a scale of 1-5 (1 = I have very low concern about this issue, 5 = I have very high concern about this issue). Reported mean of ratings for each concern (a lower average rating indicates lower concern about the issue).  ^j^AUDIT-C: Alcohol Use Disorders Identification Test – Concise screening questionnaire, 3 items (where a score of 0 indicates no alcohol use, and a score of 4+ among men and 3+ among women is considered positive for alcohol use disorder)  ^k^CES-D: Center for Epidemiological Studies Depression Scale or CES-D screening questionnaire, 10 items (where a higher score indicates greater symptoms of depression; a cut off score of ten or higher indicates the presence of significant depressive symptoms) | | | |
