# Supplementary material for: Outcomes of a Comprehensive Mobile Vaping Cessation Program in Adults Who Vape Daily: Cohort Study
Source: JMIR Form Res. 2024 Oct 28;8:e57376. doi: 10.2196/57376 (PMC11555445; doi:10.2196/57376)
Supplement: Multimedia Appendix 2 [file formative_v8i1e57376_app2.docx]

Multimedia Appendix 2. Linear regression analyses of baseline predictors of rating to setup program at 2 weeks (N=71) and number of surveys completed, number of app sessions, and number of text messages sent to coach at 6 months (N=73).

|  | **Week 2** | | | **Week 26** | | | | | | |
| --- | --- | --- | --- | --- | --- | --- | --- | --- | --- | --- |
| **Characteristic** | **N** | **Rating ease to setup program^a^** | ***P*** | **N** | **Number of surveys completed** | ***P*** | **Number of app sessions** | ***P*** | **Number of text messages sent to coach** | ***P*** |
| **Demographics** |  |  |  |  |  |  |  |  |  |  |
| Age | 71 | 0.02 (-0.02,0.07) | .41 | 73 | 0.001 (-0.04,0.04) | .97 | 1.2 (-0.8,3.2) | .26 | 0.7 (-0.09,1.6) | .08 |
| Gender^b, c^ |  |  |  |  |  |  |  |  |  |  |
| Man | 33 | 1 [Ref] |  | 33 | 1 [Ref] |  | 1 [Ref] |  | 1 [Ref] |  |
| Woman | 36 | 0.4 (-0.7,1.5) | .49 | 38 | -0.5 (-1.3,0.3) | .22 | 9.0 (-37.9,55.9) | .71 | 1.9 (-17.9,21.7) | .85 |
| Race^c^ |  |  |  |  |  |  |  |  |  |  |
| White | 56 | 1 [Ref] |  | 58 | 1 [Ref] |  | 1 [Ref] |  | 1 [Ref] |  |
| Non-white | 15 | 0.2 (-1.1,1.5) | .72 | 15 | -0.1 (-1.1,0.9) | .87 | -13.7 (-70.0,42.6) | .63 | 1.4 (-22.5,25.2) | .91 |
| Ethnicity |  |  |  |  |  |  |  |  |  |  |
| Hispanic, Latino / Latina,   or Spanish Origin | 63 | 1 [Ref] |  | 65 | 1 [Ref] |  | 1 [Ref] |  | 1 [Ref] |  |
| Not of Hispanic, Latino /   Latina, or Spanish Origin | 8 | -0.2 (-1.9,1.4) | .77 | 8 | 0.5 (-0.8,1.8) | .43 | -21.4 (-95.2,52.3) | .56 | 1.5 (-29.3,32.3) | .92 |
| Education^c^ |  |  |  |  |  |  |  |  |  |  |
| no college degree | 26 | 1 [Ref] |  | 26 | 1 [Ref] |  | 1 [Ref] |  | 1 [Ref] |  |
| 2 yr degree or greater | 45 | 0.6 (-0.5,1.7) | .31 | 47 | 0.07 (-0.8,0.9) | .88 | 54.7 (8.9,100.6) | .02 | 21.9 (2.5,41.4) | .03 |
| Income^c^ |  |  |  |  |  |  |  |  |  |  |
| Less than $50,000 | 20 | -0.1 (-1.4,1.2) | .85 | 21 | -0.4 (-1.4,0.6) | .48 | -12.7 (-68.7,43.3) | .66 | -27.5 (-50.4,-4.5) | .02 |
| $50,000 to $99,999 | 26 | 1 [Ref] |  | 26 | 1 [Ref] |  | 1 [Ref] |  | 1 [Ref] |  |
| $100,000 or more | 25 | 0.2 (-1,1.5) | .70 | 26 | -0.2 (-1.2,0.7) | .64 | -44.9 (-97.8,8.0) | .10 | -27.2 (-48.9,-5.6) | .01 |
| Employment^c^ |  |  |  |  |  |  |  |  |  |  |
| not employed | 12 | 1 [Ref] |  | 12 | 1 [Ref] |  | 1 [Ref] |  | 1 [Ref] |  |
| employed | 59 | 1.6 (0.3,3.0) | .02 | 61 | 0.06 (-1.0,1.2) | .92 | 18.4 (-42.9,79.7) | .56 | 5.6 (-20.4,31.5) | .67 |
| Self-reported health^c^ |  |  |  |  |  |  |  |  |  |  |
| Fair/Poor | 8 | 1 [Ref] |  | 8 | 1 [Ref] |  | 1 [Ref] |  | 1 [Ref] |  |
| Good | 17 | -0.8 (-2.7,1.1) | .40 | 17 | -0.7 (-2.2,0.8) | .35 | 50.4 (-31.9,132.7) | .24 | 33.4 (-0.4,67.3) | .05 |
| Excellent/Very Good | 46 | -0.6 (-2.3,1.1) | .48 | 48 | -0.4 (-1.7,0.9) | .58 | 17.4 (-56.0,90.7) | .64 | 7.5 (-22.7,37.7) | .63 |
| Smartphone |  |  |  |  |  |  |  |  |  |  |
| Android | 42 | 1 [Ref] |  | 43 | 1 [Ref] |  | 1 [Ref] |  | 1 [Ref] |  |
| iPhone | 29 | -0.6 (-1.6,0.5) | .30 | 30 | 0.3 (-0.5,1.2) | .42 | 15.9 (-30.2,62.1) | .50 | 4.1 (-15.4,23.7) | .68 |
| **Vaping and Quitting Behavior^d, e^** |  |  |  |  |  |  |  |  |  |  |
| Vape sessions per day^e^ | 71 | 0.05 (-0.04,0.1) | .28 | 73 | 0.03 (-0.09,0.04) | .45 | 2.3 (-1.5,6.1) | .24 | 0.8 (-0.8,2.4) | .32 |
| Years vaping | 71 | -0.1 (-0.3,0.1) | .35 | 73 | -0.04 (-0.2,0.1) | .60 | -5.1 (-13.0,2.8) | .21 | -0.9 (-4.3,2.5) | .61 |
| Age first tried vaping | 71 | 0.03 (-0.02,0.1) | .21 | 73 | 0.005 (-0.03,0.04) | .78 | 1.3 (-0.6,3.1) | .17 | 0.6 (-0.2,1.4) | .12 |
| Age first started thinking of  self as someone who vapes | 71 | 0.02 (-0.02,0.1) | .32 | 73 | 0.003 (-0.3,0.04) | .88 | 1.4 (-0.5,3.3) | .16 | 0.7 (-0.07,1.5) | .07 |
| Vape device used |  |  |  |  |  |  |  |  |  |  |
| Use Refillable |  |  |  |  |  |  |  |  |  |  |
| No | 44 | 1 [Ref] |  | 44 | 1 [Ref] |  | 1 [Ref] |  | 1 [Ref] |  |
| Yes | 27 | 1.3 (0.2,2.3) | .02 | 29 | -0.09 (-0.9,0.7) | .84 | -44.6 (-90.0,0.7) | .05 | -15.7 (-35.1,3.6) | .11 |
| Use Prefilled |  |  |  |  |  |  |  |  |  |  |
| No | 18 | 1 [Ref] |  | 20 | 1 [Ref] |  | 1 [Ref] |  | 1 [Ref] |  |
| Yes | 53 | -1.0 (-2.2,0.2) | .11 | 53 | -0.1 (-1.0,0.8) | .80 | 30.5 (-20.1,81.1) | .24 | 12.2 (-9.2,33.6) | .27 |
| Uses vape/e-cig flavor(s) |  |  |  |  |  |  |  |  |  |  |
| No | 14 | 1 [Ref] |  | 14 | 1 [Ref] |  | 1 [Ref] |  | 1 [Ref] |  |
| Yes | 57 | 0.4 (-0.9,1.8) | .51 | 59 | -0.7 (-1.7,0.4) | .20 | -46.9 (-103.7,9.9) | .11 | -22.4 (-46.3,1.5) | .07 |
| Nicotine type used^c^ |  |  |  |  |  |  |  |  |  |  |
| Freebase and/or   Nictotine salt | 49 | 1 [Ref] |  | 48 | 1 [Ref] |  | 1 [Ref] |  | 1 [Ref] |  |
| Don't know | 24 | 0.03 (-1.1,1.2) | .95 | 23 | 0.3 (-0.6,1.2) | .50 | 1.9 (-46.5,50.4) | .94 | -5.7 (-26.2,14.7) | .58 |
| First vape session after waking^c^ |  |  |  |  |  |  |  |  |  |  |
| 0-5 minutes | 39 | 1 [Ref] |  | 40 | 1 [Ref] |  | 1 [Ref] |  | 1 [Ref] |  |
| 6-15 minutes | 13 | -0.4 (-1.7,0.9) | .57 | 13 | -0.2 (-1.2,0.7) | .64 | 0.9 (-54.1,55.9) | .97 | 4.6 (-18.6,27.9) | .70 |
| 16 minutes or more | 19 | -0.3 (-1.6,1.0) | .66 | 20 | 0.6 (-0.4,1.6) | .26 | 20.1 (-38.6,78.8) | .50 | 9.8 (-12.7,34.6) | .44 |
| Vaping dependence via PSECDI^c^ |  |  |  |  |  |  |  |  |  |  |
| medium dependence or less | 23 | 1 [Ref] |  | 23 | 1 [Ref] |  | 1 [Ref] |  | 1 [Ref] |  |
| high dependence | 48 | -0.3 (-1.4,0.8) | .61 | 50 | 0.2 (-0.7,1.0) | .70 | 18.1 (-30.7,67.0) | .47 | 9.6 (-11.1,30.2) | .36 |
| Reasons started vaping^f, i^ |  |  |  |  |  |  |  |  |  |  |
| Boredom |  |  |  |  |  |  |  |  |  |  |
| No | 62 | 1 [Ref] |  | 64 | 1 [Ref] |  | 1 [Ref] |  | 1 [Ref] |  |
| Yes | 9 | -0.3 (-1.9,1.3) | .69 | 9 | 0.5 (-0.7,1.7) | .44 | 50.2 (-18.0,118.5) | .15 | 34.4 (6.2,62.6) | .02 |
| Curiosity |  |  |  |  |  |  |  |  |  |  |
| No | 48 | 1 [Ref] |  | 50 | 1 [Ref] |  | 1 [Ref] |  | 1 [Ref] |  |
| Yes | 23 | -0.9 (-2,0.2) | .13 | 23 | 0.7 (-0.1,1.6) | .10 | -32.3 (-80.7,16.2) | .19 | -13.0 (-33.5,7.5) | .21 |
| Flavor choices |  |  |  |  |  |  |  |  |  |  |
| No | 59 | 1 [Ref] |  | 61 | 1 [Ref] |  | 1 [Ref] |  | 1 [Ref] |  |
| Yes | 12 | 0.9 (-0.5,2.3) | .22 | 12 | -1.0 (-2.0,0.1) | .08 | -53.7 (-113.9,6.5) | .08 | -23.6 (-49.0,1.8) | .07 |
| Lack of odor |  |  |  |  |  |  |  |  |  |  |
| No | 59 | 1 [Ref] |  | 61 | 1 [Ref] |  | 1 [Ref] |  | 1 [Ref] |  |
| Yes | 12 | 1.0 (-0.4,2.4) | .17 | 12 | -0.06 (-1.2,1.0) | .92 | 47.7 (-12.7,108.1) | .12 | 5.2 (-20.8,31.1) | .70 |
| My friends/peer group vape |  |  |  |  |  |  |  |  |  |  |
| No | 47 | 1 [Ref] |  | 49 | 1 [Ref] |  | 1 [Ref] |  | 1 [Ref] |  |
| Yes | 24 | 0.5 (-0.7,1.6) | .42 | 24 | 0.7 (-0.1,1.6) | .09 | -2.5 (-50.9,46.0) | .92 | -11.8 (-32.1,8.5) | .25 |
| To help me quit smoking |  |  |  |  |  |  |  |  |  |  |
| No | 30 | 1 [Ref] |  | 30 | 1 [Ref] |  | 1 [Ref] |  | 1 [Ref] |  |
| Yes | 41 | -0.7 (-1.7,0.4) | .22 | 43 | -0.002 (-0.8,0.8) | .99 | 29.8 (-16.0,75.6) | .20 | 16.8 (-2.4,36.0) | .09 |
| To manage stress |  |  |  |  |  |  |  |  |  |  |
| No | 51 | 1 [Ref] |  | 52 | 1 [Ref] |  | 1 [Ref] |  | 1 [Ref] |  |
| Yes | 20 | 0.4 (-0.7,1.6) | .46 | 21 | -0.8 (-1.7,0.09) | .08 | 71.7 (24.2,119.3) | .003 | 18.6 (-2.3,39.4) | .08 |
| Because I can vape/use e- cigs indoors |  |  |  |  |  |  |  |  |  |  |
| No | 53 | 1 [Ref] |  | 55 | 1 [Ref] |  | 1 [Ref] |  | 1 [Ref] |  |
| Yes | 18 | -0.7 (-1.9,0.5) | .27 | 18 | 0.1 (-0.8,1.1) | .80 | 0.8 (-52.0,53.6) | .98 | 1.3 (-21.1,23.6) | .91 |
| Reasons continued to vape^g^ |  |  |  |  |  |  |  |  |  |  |
| It was too difficult to quit |  |  |  |  |  |  |  |  |  |  |
| No | 26 | 1 [Ref] |  | 28 | 1 [Ref] |  | 1 [Ref] |  | 1 [Ref] |  |
| Yes | 45 | -0.8 (-1.8,0.3) | .16 | 45 | 0.5 (-0.4,1.3) | .27 | -9.3 (-56.0,37.5) | .70 | -14.3 (-33.9,5.2) | .15 |
| Boredom |  |  |  |  |  |  |  |  |  |  |
| No | 61 | 1 [Ref] |  | 62 | 1 [Ref] |  | 1 [Ref] |  | 1 [Ref] |  |
| Yes | 10 | -0.6 (-2.1,1) | .47 | 11 | -0.1 (-1.3,1) | .84 | 5.1 (-58.5,68.8) | .87 | 25.2 (-1.1,51.5) | .06 |
| Flavor choices |  |  |  |  |  |  |  |  |  |  |
| No | 56 | 1 [Ref] |  | 58 | 1 [Ref] |  | 1 [Ref] |  | 1 [Ref] |  |
| Yes | 15 | 0.9 (-0.4,2.2) | .16 | 15 | 0.001 (-1.0,1.0) | .99 | -17.1 (-73.4,39.1) | .55 | -12.8 (-36.5,10.8) | .29 |
| Lack of odor |  |  |  |  |  |  |  |  |  |  |
| No | 62 | 1 [Ref] |  | 63 | 1 [Ref] |  | 1 [Ref] |  | 1 [Ref] |  |
| Yes | 9 | -0.3 (-1.9,1.3) | .69 | 10 | -1.0 (-2.2,0.2) | .09 | -21.8 (-87.8,44.3) | .52 | -4.3 (-32.3,23.7) | .76 |
| My friends/peer group vape |  |  |  |  |  |  |  |  |  |  |
| No | 59 | 1 [Ref] |  | 61 | 1 [Ref] |  | 1 [Ref] |  | 1 [Ref] |  |
| Yes | 12 | -1.0 (-2.4,0.3) | .14 | 12 | 0.5 (-0.6,1.6) | .33 | 6.1 (-55.3,67.6) | .84 | -5.7 (-31.6,20.3) | .67 |
| To help me quit   smoking/stay quit |  |  |  |  |  |  |  |  |  |  |
| No | 43 | 1 [Ref] |  | 43 | 1 [Ref] |  | 1 [Ref] |  | 1 [Ref] |  |
| Yes | 28 | 0.2 (-0.9,1.3) | .74 | 30 | -0.4 (-1.2,0.4) | .35 | 0.02 (-46.3,46.3) | .99 | 12.5 (-6.9,31.8) | .21 |
| To manage stress |  |  |  |  |  |  |  |  |  |  |
| No | 37 | 1 [Ref] |  | 39 | 1 [Ref] |  | 1 [Ref] |  | 1 [Ref] |  |
| Yes | 34 | -0.3 (-1.3,0.8) | .63 | 34 | 0.06 (-0.7,0.9) | .88 | 33.2 (-11.8,78.2) | .15 | 11.1 (-8.0,30.3) | .25 |
| Because I can vape/use e-  cigs indoors |  |  |  |  |  |  |  |  |  |  |
| No | 52 | 1 [Ref] |  | 50 | 1 [Ref] |  | 1 [Ref] |  | 1 [Ref] |  |
| Yes | 21 | 0.3 (-0.9,1.4) | .64 | 21 | -0.3 (-1.1,0.6) | .58 | 4.3 (-46.0,54.6) | .87 | 3.4 (-17.9,24.6) | .76 |
| Quit attempts in the past 12   months | 71 | -0.4 (-0.6,-0.1) | .003 | 73 | 0.1 (-0.06,0.3) | .16 | 1.6 (-9.8,13.0) | .79 | 0.3 (-4.6,5.1) | .92 |
| Methods used in past vaping  quit attempts |  |  |  |  |  |  |  |  |  |  |
| Cold Turkey |  |  |  |  |  |  |  |  |  |  |
| No | 32 | 1 [Ref] |  | 34 | 1 [Ref] |  | 1 [Ref] |  | 1 [Ref] |  |
| Yes | 39 | -0.5 (-1.5,0.6) | .40 | 39 | 0.3 (-0.5,1.1) | .52 | 18.6 (-26.8,64.0) | .42 | -1.0 (-20.3,18.3) | .92 |
| Taper - decrease number of   vape sessions per day, puffs  per session, and/or the   length |  |  |  |  |  |  |  |  |  |  |
| No | 38 | 1 [Ref] | .98 | 39 | 1 [Ref] | .77 | 1 [Ref] | .48 | 1 [Ref] | .95 |
| Yes | 33 | -0.02 (-1.1,1.0) |  | 34 | 0.1 (-0.7,0.9) |  | -16.2 (-61.7,29.3) |  | 0.6 (-18.7,19.9) |  |
| Taper - decrease nicotine   concentration in my vape   juice/e-liquid |  |  |  |  |  |  |  |  |  |  |
| No | 47 | 1 [Ref] |  | 48 | 1 [Ref] |  | 1 [Ref] |  | 1 [Ref] |  |
| Yes | 24 | -0.05 (-1.2,1.1) | .93 | 25 | -0.5 (-1.3,0.4) | .28 | -20.3 (-68.1,27.4) | .40 | -7.2 (-27.5,13.0) | .48 |
| Medication - Nicotine   Replacement Therapy   (NRT) |  |  |  |  |  |  |  |  |  |  |
| No | 63 | 1 [Ref] |  | 65 | 1 [Ref] |  | 1 [Ref] |  | 1 [Ref] |  |
| Yes | 8 | -0.6 (-2.3,1.1) | .48 | 8 | 0.5 (-0.8,1.8) | .48 | 20.6 (-52.1,93.3) | .58 | -9.4 (-40.1,21.4) | .55 |
| Any medication |  |  |  |  |  |  |  |  |  |  |
| No | 61 | 1 [Ref] |  | 63 | 1 [Ref] |  | 1 [Ref] |  | 1 [Ref] |  |
| Yes | 10 | -0.4 (-2.0,1.1) | .56 | 10 | 0.5 (-0.7,1.7) | .40 | 29.3 (-36.5,95.2) | .38 | -1.6 (-29.6,26.4) | .91 |
| None |  |  |  |  |  |  |  |  |  |  |
| No | 53 | 1 [Ref] |  | 54 | 1 [Ref] |  | 1 [Ref] |  | 1 [Ref] |  |
| Yes | 18 | -0.2 (-1.4,1.0) | .71 | 19 | -0.3 (-1.3,0.6) | .46 | 9.2 (-42.6,61.1) | .73 | 7.1 (-14.8,29.0) | .52 |
| Reasons to stop vaping^h^ |  |  |  |  |  |  |  |  |  |  |
| Financial Cost |  |  |  |  |  |  |  |  |  |  |
| No | 15 | 1 [Ref] |  | 16 | 1 [Ref] |  | 1 [Ref] |  | 1 [Ref] |  |
| Yes | 56 | -0.2 (-1.4,1.1) | .82 | 57 | -0.1 (-1.1,0.9) | .80 | -18.8 (-73.6,36.1) | .50 | 5.4 (-17.8,28.6) | .65 |
| Freedom from addiction |  |  |  |  |  |  |  |  |  |  |
| No | 11 | 1 [Ref] |  | 12 | 1 [Ref] |  | 1 [Ref] |  | 1 [Ref] |  |
| Yes | 60 | 0.9 (-0.6,2.3) | .24 | 61 | 0.06 (-1.0,1.2) | .92 | 17.7 (-43.6,79.0) | .57 | 4.6 (-21.4,30.6) | .73 |
| Social impacts |  |  |  |  |  |  |  |  |  |  |
| No | 41 | 1 [Ref] |  | 41 | 1 [Ref] |  | 1 [Ref] |  | 1 [Ref] |  |
| Yes | 30 | -0.03 (-1.1,1.0) | .96 | 32 | 0.06 (-0.8,0.9) | .88 | 7.7 (-38.2,53.5) | .74 | 19.8 (1.0 -38.7) | .04 |
| Attitudes Towards Quitting   Vaping |  |  |  |  |  |  |  |  |  |  |
| DTQ | 71 | -0.05 (-0.2,0.1) | .61 | 73 | 0.2 (0.01,0.3) | .04 | -6.7 (-14.6,1.3) | .10 | -1.8 (-5.2,1.6) | .29 |
| STQ | 71 | -0.001 (-0.2,0.2) | .99 | 73 | 0.05 (-0.1,0.2) | .50 | -6.3 (-14.0,1.3) | .11 | -2.4 (-5.7,0.8) | .15 |
| How is vaping/using e-cigs   and drinking alcohol linked for   you^c^ |  |  |  |  |  |  |  |  |  |  |
| linked | 30 | 1 [Ref] |  | 31 | 1 [Ref] |  | 1 [Ref] |  | 1 [Ref] |  |
| not linked | 16 | 0.3 (-1.0,1.7) | .65 | 16 | -0.4 (-1.4,0.7) | .51 | 38.3 (-20.9,97.6) | .20 | 12.5 (-12.6,37.7) | .33 |
| do not drink | 25 | 0.8 (-0.4,2.0) | .20 | 26 | 0.2 (-0.7,1.1) | .72 | 8.5 (-42.6,59.7) | .74 | 0.5 (-21.2,22.3) | .96 |
| Alcohol use behavior via   AUDIT-C | 71 | -0.1 (-0.3,0.1) | .31 | 73 | -0.1 (-0.3,0.08) | .27 | 2.8 (-7.1,12.7) | .58 | 1.1 (-3.1,5.3) | .60 |
| Presence of depressive   symptoms via CES-D | 71 | -0.03 (-0.1,0.1) | .49 | 73 | 0.03 (-0.03,0.09) | .36 | -2.4 (-6.0,1.1) | .18 | -0.4 (-1.9,1.1) | .62 |
| **Current Product and Medication Use^i, j^** |  |  |  |  |  |  |  |  |  |  |
| Other vape juice/e-liquid   products used^c^ |  |  |  |  |  |  |  |  |  |  |
| None | 58 | 1 [Ref] |  | 60 | 1 [Ref] |  | 1 [Ref] |  | 1 [Ref] |  |
| CBD, THC, or Other | 13 | 0.2 (-1.1,1.6) | .75 | 13 | -0.1 (-1.2,1) | .86 | 17.2 (-42.2,76.5) | .57 | 1.5 (-23.6,26.7) | .91 |
| **Former Smoking-Related Measures^k^** |  |  |  |  |  |  |  |  |  |  |
| Former smoker |  |  |  |  |  |  |  |  |  |  |
| No | 20 | 1 [Ref] |  | 20 | 1 [Ref] |  | 1 [Ref] |  | 1 [Ref] |  |
| Yes | 51 | -0.5 (-1.7,0.7) | .39 | 53 | -0.6 (-1.5,0.3) | .19 | 19.3 (-31.6,70.1) | .46 | 16.0 (-5.3,37.2) | .14 |
| **Living and Social Environment^l^** |  |  |  |  |  |  |  |  |  |  |
| Of the adults you live with, how many vape/use e-cigs | 71 | -1.1 (-2.1,-0.2) | .02 | 73 | -0.4 (-1.1,0.4) | .36 | -22.4 (-65.1,20.2) | .30 | -9.0 (-27.0,9.1) | .33 |
| Lives with a romantic partner   who vapes/uses e-cigs |  |  |  |  |  |  |  |  |  |  |
| No | 56 | 1 [Ref] |  | 58 | 1 [Ref] |  | 1 [Ref] |  | 1 [Ref] |  |
| Yes | 15 | -0.9 (-2.1,0.4) | .18 | 15 | -0.7 (-1.7,0.3) | .19 | -33.6 (-89.4,22.2) | .24 | -21.2 (-44.5,2.1) | .07 |
| Number of close friends who   vape/use e-cigs | 71 | -0.1 (-0.2,0.03) | .16 | 73 | 0.03 (-0.06,0.1) | .56 | 5.4 (0.7,10.1) | .03 | 0.9 (-1.1,3.0) | .38 |
| Of the adults you live with,   how many smoke cigarettes | 71 | -0.5 (-2.0,1.0) | .54 | 73 | 0.2 (-0.9,1.4) | .67 | 4.1 (-60.3,68.5) | .90 | 2.0 (-25.2,29.2) | .89 |
| Number of close friends who  smoke cigarettes | 71 | 0.1 (-0.1,0.2) | .54 | 73 | 0.05 (-0.08,0.2) | .45 | 14.6 (8.1,21.0) | <.001 | 2.9 (-0.1,5.9) | .06 |
| How much would quitting   vaping positively influence  others in your “online” world,   scale 1-10 | 71 | -0.1 (-0.3,0.03) | .10 | 73 | 0.07 (-0.05,0.2) | .24 | 4.6 (-2.2,11.4) | .18 | 2.3 (-0.5,5.2) | .11 |
| How much would quitting   vaping positively influence   others in your “real” world,   scale 1-10 | 71 | 0.003 (-0.2,0.2) | .97 | 73 | -0.02 (-0.14,0.1) | .72 | 4.3 (-2.4,11.0) | .21 | 1.4 (-1.5,4.2) | .34 |

^a^  How easy or difficult was it to set-up and get started in the Pivot program? (1 = Very difficult, 10 = Very easy); 71 responded at 2 weeks

^b^  Two participants reported Prefer to self-describe representing < 10% of respondents and therefore not included.

^c^ Category collapsed.

^d^ Nicotine concentration (mg/mL), Amount of nicotine (mg) used per week, Vape juice/e-liquid volume of primary device are not included as these were only answered in the context of Refillable and Prefilled vape use. Question not posed to all participants.

^e^ Ratings related to question “Please rate each issue as it relates to challenges you may face when quitting vaping/e-cigs” for Weight gain, Expected anxiety and/or emotional discomfort, Expected physical discomfort, Changes in friend or family relationships, are not included in analysis as these questions only presented to the non-smokers.

^f^ Reasons started vaping: My family members vape, Social image, and Other were not analyzed each had a group representing < 10% of respondents and therefore not included.

^g^ Reasons continued to vape: My family members vape, Social image, and Other each had a group representing < 10% of respondents and therefore not included.

^h^ Reasons to stop vaping: Health had a group representing < 10% of respondents and therefore not included.

^i^ Tobacco products used not included as all 73 was using E-cigarettes/vaping and 1 using Hookah representing < 10% of respondents and therefore not included

^j^ Medications currently taking: Yes representing < 10% of respondents and therefore not included

^k^ All questions answered only by those smoking not included as questions were not posed to all participants**.**

^l^ Response to Lives with a romantic partner who smokes cigarettes: Yes represents < 10% of respondents and therefore not included

All variables with *P*<.05 significance from univariate regressions underwent stepwise regression using significance level *P*<.05 to enter and remain in the final model. Final models are presented below.

| **Response** | **N** | **R^2^** | **Variables** | **Coefficient** | **95% CI** | ***P*** |
| --- | --- | --- | --- | --- | --- | --- |
| Rating ease to setup program^a^ | 71 | 0.19 | Employment – employed vs   not employed | 1.7 | (0.4,3.0) | .01 |
|  |  |  | Quit attempts in the past 12 months | -0.4 | (-0.6,-0.1) | .003 |
| Number of surveys completed | 73 | 0.06 | Attitudes Towards Quitting Vaping  DTQ^d^ | 0.2 | (0.01,0.3) | .04 |
| Number of app sessions^b^ | 73 | 0.32 | Education - 2 yr degree or greater vs   no college degree | 46.1 | (6.6,85.5) | .02 |
|  |  |  | Reasons started vaping: To manage stress  Yes vs No | 49.9 | (7.3,92.4) | .02 |
|  |  |  | Number of close friends who smoke   cigarettes | 12.8 | (6.8,18.9) | <.001 |
| Number of text messages sent to coach^c^ | 73 | 0.25 | Education - 2 yr degree or greater vs   no college degree | 21.9 | (3.7,40.2) | .02 |
|  |  |  | Income - Less than $50,000 vs   $50,000 to $99,999 | -33.5 | (-43.9,-0.9) | .04 |
|  |  |  | Income - $100,000 or more vs  $50,000 to $99,999 | -22.4 | (-53.6,-13.6) | .001 |
|  |  |  | Reasons started vaping: Boredom  Yes vs No | 39.8 | (14.1,65.5) | .002 |

^a^ Not included in final model per stepwise regression: Use Refillable device Yes vs No, and Num adults you live with, how many vape/use e-cigs

^b^ Not included in final model per stepwise regression: Number of close friends who vape/use e-cigs

^c^ Not included in final model per stepwise regression: Reason to stop vaping = Social impact – Yes vs No

^d^ DTQ difficulty to stay quit; “If you were to quit vaping/e-cigs right now, how difficult do you think it would be to stay vape free?
(1=Really difficult to stay vape/e-cig free; 10=Really easy to stay vape/e-cig)”
